# Supplementary material for: Sustainable intensification for a larger global rice bowl
Source: Nat Commun. 2021 Dec 9;12:7163. doi: 10.1038/s41467-021-27424-z (PMC8660894; doi:10.1038/s41467-021-27424-z)
Supplement: Supplementary file 1 — Supplementary Information [file 41467_2021_27424_MOESM1_ESM.pdf]

Supplementary Information for "Sustainable intensification for a larger global rice bowl" by Yuan et al.

CONTENTS

Supplementary Methods

1. **Estimation of yield gaps …………………………………………………….. 2**
2. **Estimation of greenhouse gas emissions and energy inputs ………………. 3**
3. **Estimation of nitrogen balance ……………………………………...…. 6**
4. **Pesticide application and toxicity …………………………...……………. 7**
5. **Estimation of labor inputs ……………………………………………….... 7**

Supplementary Figs. 1-11 ……………………………………………………….. 9-19

Supplementary Tables 1-14 …………………………………………………... 20-34

Additional References associated with Supplementary Information ………… 35-45

Supplementary methods

1. Estimation of yield gaps

Estimates of yield potential (or water-limited yield potential for rainfed rice) for each rice crop cycle in each of the 32 rice cropping systems were retrieved from the Global Yield Gap Atlas (www.yieldgap.org) and best alternative publications for Australia (AUIS) where no values were reported by GYGA[1,2] (Supplementary Table 7). Estimates of yield potential in GYGA followed these main steps: (1) selection of representative climate zones based on dominant crop areas, (2) selection of reference weather stations (RWS) buffer that represent the selected CZs, (3) selection of dominant soil types and cropping systems in a 100 km radius around the RWS buffer, and (4) crop model simulations to establish rainfed or irrigated yield potential[3]. For each buffer-year-water regime combination, each rice crop cycle (in each cropping system) x soil type combination was simulated, and then weighted by their relative proportion to retrieve an average yield potential for each buffer. Yield potential simulation in GYGA was performed using the crop growth and development model ORYZA2000 or ORYZA (v3) (except for APSIM in the case of India) and based on best available source daily weather data (giving preference to measured weather) and local soils and crop calendar and for the most representative rice varieties planted in each region[1,3,4].

For estimating yield potential for each of 32 rice cropping systems, yield potential (or water-limited yield potential for rainfed rice) of each rice crop cycle in each cropping system was retrieved from GYGA for available years for the major rice-producing buffers within the region where the data on this specific rice cropping system come from[1]. Then for each rice crop cycle, data on yield potential were averaged across years and buffers to represent yield potential of the corresponding rice crop cycle in each system (Supplementary Fig. 3). The coefficient of variation (CV) of yield potential (or water-limited yield potential for rainfed rice) across years was determined for each cropping system, and was plotted against average yield (% of potential) (Supplementary Fig. 4). It was noted that CV of yield potential of irrigated rice in Australia (AUIS) was assumed to be equal to that of actual yield, as there was only average yield potential reported in Lacy et al[2]. The yield gap was calculated as the difference between yield potential (or water-limited yield potential for rainfed rice) and the 3-y average actual yield. For cropping systems including more than one rice crop cycle, the average yield potential for rice was

estimated by averaging yield potential across rice crop cycles based on harvested rice area. Average yield and yield gap were expressed as % of the yield potential (Fig. 1 and Supplementary Fig. 5).

2. Estimation of greenhouse gas emissions and energy inputs

The CO_2, methane (CH_4), and nitrous oxide (N_2O) emissions were estimated for each rice crop. Emissions from various agricultural inputs were calculated by multiplying input amount by corresponding emission factor for each input and summing emissions across inputs in a rice crop (Supplementary Table 10). In the case of fossil fuel used for field operations, it was calculated based on the number and type of farm operations and associated fuel requirements (Supplementary Table 11).

The CH_4 emissions from rice paddy field were calculated following Intergovernmental Panel on Climate Change (IPCC) methodology[5], as follows:

$$CH_4\ emission\ (kg\ CH_4\ ha^{-1}) = T \times EF_C \times SF_W \times SF_P \times SF_O$$

where T is rice cultivation period for each rice cycle, which was derived from the reported crop establishment and harvest dates; EF_C is a baseline emission factor for continuously flooded fields without organic amendments; SF_W and SF_P are scaling factors to account for differences in water regime during the cultivation period and during the pre-season before the cultivation period, respectively, and SF_O is a scaling factor which varies for both type and amount of organic amendment applied (*e.g.*, straw, manure, compost) and is calculated as follows:

$$SF_O = \left(1 + \sum_i ROA_i \times CFOA_i\right)^{0.59}$$

where ROA_i is the application rate of organic amendment i; $CFOA_i$ is the conversion factor for organic amendment i.

Total N_2O emissions were calculated as the sum of direct and indirect N_2O emissions. Following van Groenigen et al.[6], direct soil N_2O emissions for a given rice cycle were calculated based on the magnitude of the N surplus as:

$$N_2O\ emissions\ (kg\ N_2O - N\ ha^{-1}) = 1.435 + 0.081 \times e^{0.0443 \times N-surplus}$$

where N surplus is calculated as the total applied N input from fertilizers and manures minus plant N accumulated in the crop (grain plus residue) at physiological maturity.

Indirect N_2O emissions were estimated based on the IPCC methodology[7], assuming indirect N_2O emissions represent 20% of direct N_2O emissions.

All emissions were converted to CO_2-eq (also called GWP), with GWP for CH_4 set at 25 relatives to CO_2 and for N_2O set at 298 on a per mass basis over a 100-year time horizon[8]. For each rice crop in each of 32 rice cropping systems, GWP (kg of CO_2-eq) was calculated as the sum of CO_2, CH_4, and N_2O emissions expressed as CO_2-eq. For cropping systems including more than one rice crop, the GWP for rice cropping system on a per crop basis (kg CO_2-eq ha^{-1} $crop^{-1}$) was estimated by averaging the GWP across rice crops (Fig. 2), and total GWP for the cropping system on an annual basis (kg CO_2-eq ha^{-1}) was calculated by summing CO_2-eq across crops (Supplementary Fig. 6). Yield-scaled GWP (kg CO_2-eq Mg^{-1} of rice grain) was calculated as the quotient between GWP and grain yield for each of the 32 cropping systems[9] (Fig. 2).

Across the 32 cropping systems, major contributors to GWP are CH_4 emissions from rice growing in lowland systems with soils kept purposely flooded (50%), emissions associated with manufacturing, packaging, and transportation of agricultural inputs (31%), and soil N_2O emissions derived from N application (19%). Variation in CH_4 emissions across cropping systems is mostly associated with differences in water and straw management and length of the cropping season cycle, from field preparation to harvest. In the case of upland rice production in Brazil, rice is grown in aerobic (non-flooded) soil conditions, which reduces CH_4 emissions and GWP (Fig. 2A, B). In contrast, major drivers for differences in CH_4 emissions across flooded-rice systems are length of the rice crop growing cycle and straw management (Supplementary Fig. 1 and Supplementary Table 5). Cropping systems where straw is left in the field and/or with long crop cycle length (e.g., Australia) have higher CH_4 emissions and GWP, on a per-crop basis, than systems where crop residues are removed from the field and/or with shorter duration of the rice crop growing cycle (e.g., Indonesia). The positive effect of shorter crop cycle length at reducing CH_4 emissions is not apparent on an annual basis because short crop cycle length is associated with tropical rice systems, which, in turn, have a higher number of rice crops per year.

Similar to GHG emissions invention, fossil-fuel energy input was calculated based on input rates and their associated embodied energy associated with their manufacturing, packaging, and

transportation (Supplementary Table 12). Energy input from labor was estimated by multiplying labor requirement by energy cost of agricultural labor. Energy input from machinery associated with field operation was estimated based on an embodied energy value of 125.4 MJ kg^{-1} and assumptions on machinery size as proposed by Stout[10], machinery lifespan of 10 years[11], and machinery working time in each field operation (Supplementary Table 11). Energy inputs from diesel fuel consumed in mechanical field operations including tillage, rice planting, fertilizing, spraying, weeding, and harvesting was calculated based on type and number of field operations per rice cycle and associated fuel requirement (Supplementary Table 11). Rice grain threshing is operated manually in countries where grain is reported to be harvested manually, so there is no additional diesel requirement in grain threshing in these cases.

Irrigation is via canal without the need of pumping in most cases, but irrigation water is pumped in some other rice-cropping systems in regions such as southern Brazil, China, and Uruguay (Supplementary Table 4). For these cases, energy use from diesel or electricity for irrigation pumping was estimated based on applied irrigation volume, percentage of pumping for irrigation, energy source, and water depth. Average operating pressure and pumping efficiency were assumed to be 30 psi and 80%, respectively, which are considered typical values for farmer-owned pumping plants[12]. Irrigation pumps are normally powered by diesel or electric engines. Diesel and electric motor efficiency of 40% and 90% were assumed, respectively[13].

Drying of grain is operated with traditional drying system (e.g., sun drying, field drying and stacking) in most rice-producing countries or regions, while rice grain is exposed to artificial drying in Australia, Brazil, Uruguay, and the USA. Grain drying process is assumed to be fueled by LPG[14]. LPG use for grain drying in these cases was calculated by considering that rice grain is harvested at a moisture content of 200 g H_2O kg^{-1} fresh weight and it is artificially dried to a moisture content of 130 g H_2O kg^{-1} to enable long storage with minimal losses[15]. Energy use for grain drying was estimated by assuming that energy input needed by a conventional dryer is 5 MJ kg^{-1} of removed water[16]. LPG usage during grain drying process was calculated as the ratio between energy use in grain drying and embodied energy per liter of LPG (25.6 MJ l^{-1})[17].

For each rice crop cycle in each of 32 rice cropping systems, energy input rate (GJ ha^{-1}) was calculated as the sum of fossil-fuel energy inputs (including labor input). For cropping systems including more than one rice crop, the energy input for rice cropping system on a per-crop basis

(GJ ha^{-1} $crop^{-1}$) was estimated by averaging the energy input across rice crops, and total energy input for the system on an annual basis (GJ ha^{-1} y^{-1}) was calculated by summing energy input across the rice crops (Supplementary Fig. 11). Similarly, net energy yield was calculated as the difference between energy output and input (GJ ha^{-1})[18], which was expressed on both per-crop and annual basis (Supplementary Fig. 11).

There was a strong correlation between energy input and GWP on both per-crop (r=0.81; p<0.01) and annual basis (r=0.92; p<0.01) at a global scale, so we are only showing GWP in the main text to avoid redundancy. The relationship between average yield (expressed as % of potential) and energy input as well as net energy yield on both per-crop and annual basis are shown in the Supplementary Fig. 11.

3. Estimation of nitrogen balance

Nitrogen (N) balance was calculated as the external N input including from synthetic N fertilizer, manure, and biological N fixation minus N removal with the harvested grain (and straw if it was burned or removed out of field) following Dobermann and Witt[19]. The N input and N removal were estimated for each rice crop cycle. The N input via manure was calculated based on the amount and source of manure and average N concentration; the latter was assumed to be 0.6% and 0.3% for animal manure and plant compost, respectively[20,21]. We used a global mean value for biological N fixation in lowland rice fields of 30 kg N ha^{-1} $crop^{-1}$ [22,23]; biological N fixation in upland rice was assumed to represent 10% of that in lowland rice[24]. The N inputs from atmospheric deposition and irrigation water or precipitation were not available, and they were not considered in our estimates as N input from these two sources would almost be offset by N losses through leaching, lixiviation, and denitrification[21].

Grain N removal was calculated based on the average grain yield and rice grain N concentration of 1.06%[25]. The removal of N from crop residues was estimated from the total removal assuming (1) a typical fraction of straw remaining in the field in different straw managements (left in field, burn, or remove out of field), (2) a typical fraction of N lost from the crop residues in each of these three situations, and (3) a typical rice residue N concentration of 0.63%[21,25] (Supplementary Table 13). The production of rice residue was estimated by multiplying grain yield by an assumed average grain-to-straw ratio of 1.0[26].

The N balance was estimated for each rice crop cycle. For cropping systems including more than one rice crop, N balance for each rice cropping system on a per-crop basis (kg N ha^{-1} $crop^{-1}$) was estimated by averaging the N balance across rice crops (Fig. 3), and N balance for the system on an annual basis (kg N ha^{-1}) was calculated by summing the N balance across crops (Supplementary Fig. 7). The yield-scaled N balance was estimated as the quotient between N balance and grain yield, and expressed as kg of N per Mg of rice grain (Fig. 3).

4. Pesticide application and toxicity

Number of pesticide applications (including insecticide, herbicide, and fungicide) was used to measure the environmental impact associated with pesticides use in rice production. We also assessed the toxicity level by calculating the amount of active ingredient applied per hectare and by estimating Environmental Impact Quotient (EIQ) following Kovach et al. environmental risk assess methodology[27]. For each pesticide item in a rice crop cycle, EIQ was calculated by multiplying the quantity of this pesticide in active ingredient (a.i.) by the corresponding EIQ index. Total EIQ for a rice crop cycle was the sum of EIQ corresponding to each pesticide usage.

There is a significant and positive relationship between the two toxicity level indices (pesticide application rate in a.i. and EIQ per hectare) on a per-crop basis (r=0.96; p<0.01) as EIQ is based on a.i. amount. The EIQ was also significantly and positively correlated with number of pesticides application on a per-crop basis (r=0.87; p<0.01). However, there might be some uncertainties in EIQ estimation associated with sketchy reporting of pesticide products and application rate, and there was considerable variation in the reliability of such data among countries or regions. Therefore, number of pesticide applications, instead of toxicity, was used to evaluate environmental impact. Yield-scaled number of pesticide applications (expressed as number of pesticide applications per Mg of rice grain) was also estimated (Fig. 2).

5. Estimation of labor inputs

Labor input involved in land preparation, seed preparation, crop establishment, water irrigation, fertilization, pesticide application, weeding, harvesting, threshing, and drying was collected for each rice crop cycle in each of 32 rice cropping systems (Fig. 4 and Supplementary Table 4).

Rice crops can be either direct seeded or transplanted. Seeds are sown directly in the field in direct-seeded rice, while in transplanted rice, seedlings are first raised in seedbeds before they are planted in the field. Direct seeded rice is less labor-intensive as compared with transplanted rice[28,29]. Therefore, we analyzed labor input of each cropping systems considering the crop establishment methods.

The degree of mechanization (high, intermediate, and low) was based on the degree to which the different on-farm operations were mechanized or manual, including land preparation, sowing or transplanting, fertilization, pesticide application, weeding, harvesting, threshing, and grain drying. For cropping systems including more than one rice crop cycle, the total labor input was estimated by summing the labor input across rice crops (Fig. 4 and Supplementary Fig. 6). Yield-scaled labor (expressed as number of hours per Mg of rice grain) was calculated for each of 32 rice cropping systems (Fig. 4).

Supplementary Figures

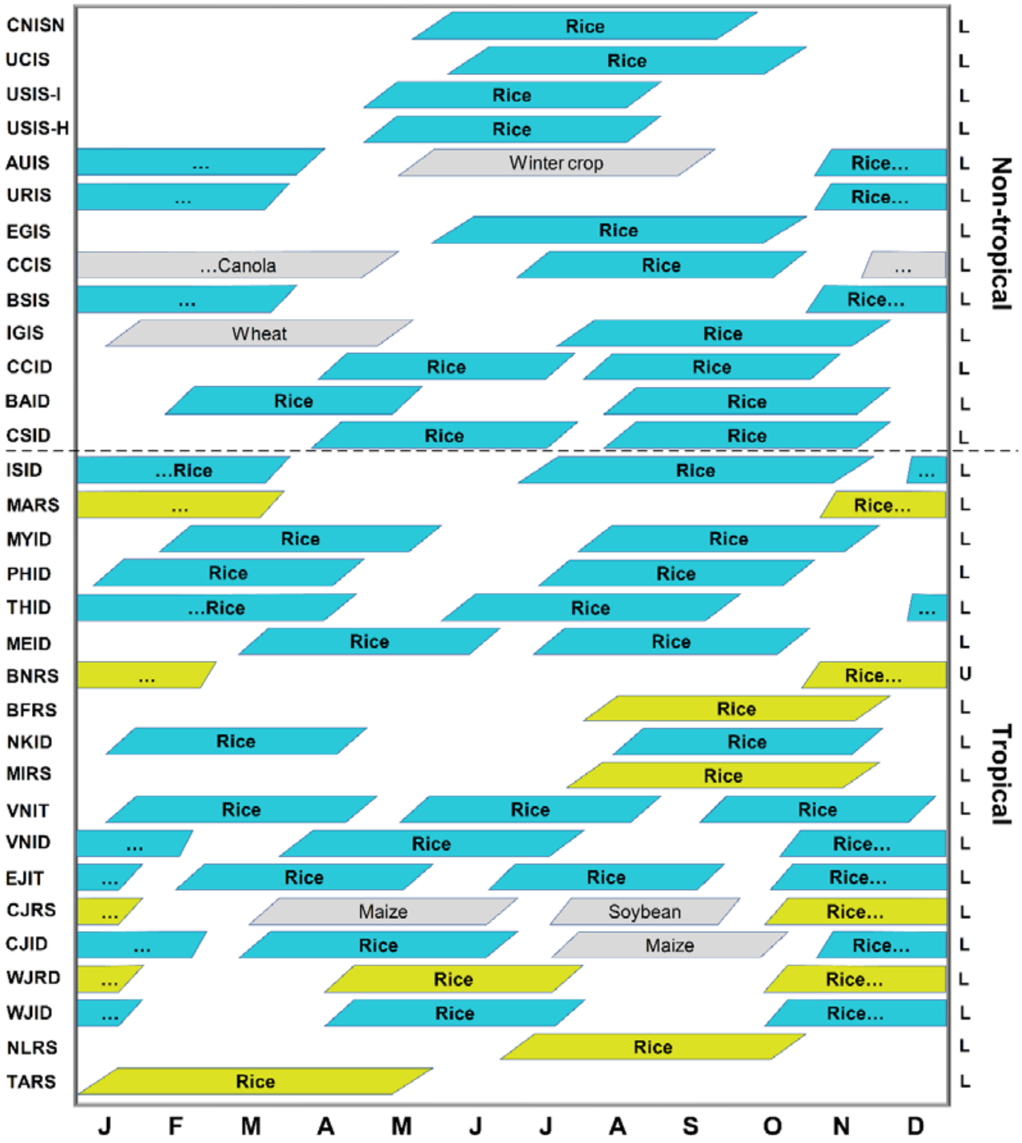

Supplementary Figure 1. Crop calendars for 32 rice cropping systems. Cropping systems were sorted according to latitude, from non-tropical (top) to tropical regions (bottom). Each box represents a crop cycle, from establishment (either transplanting or direct seeding) to harvest maturity. Colors indicate water regime: irrigated (blue) or rainfed (yellow). Letters on the right axis indicate ecosystem: lowland (L) or upland (U). Cropping system codes are shown in Supplementary Table 2. Data are provided in Source Data.

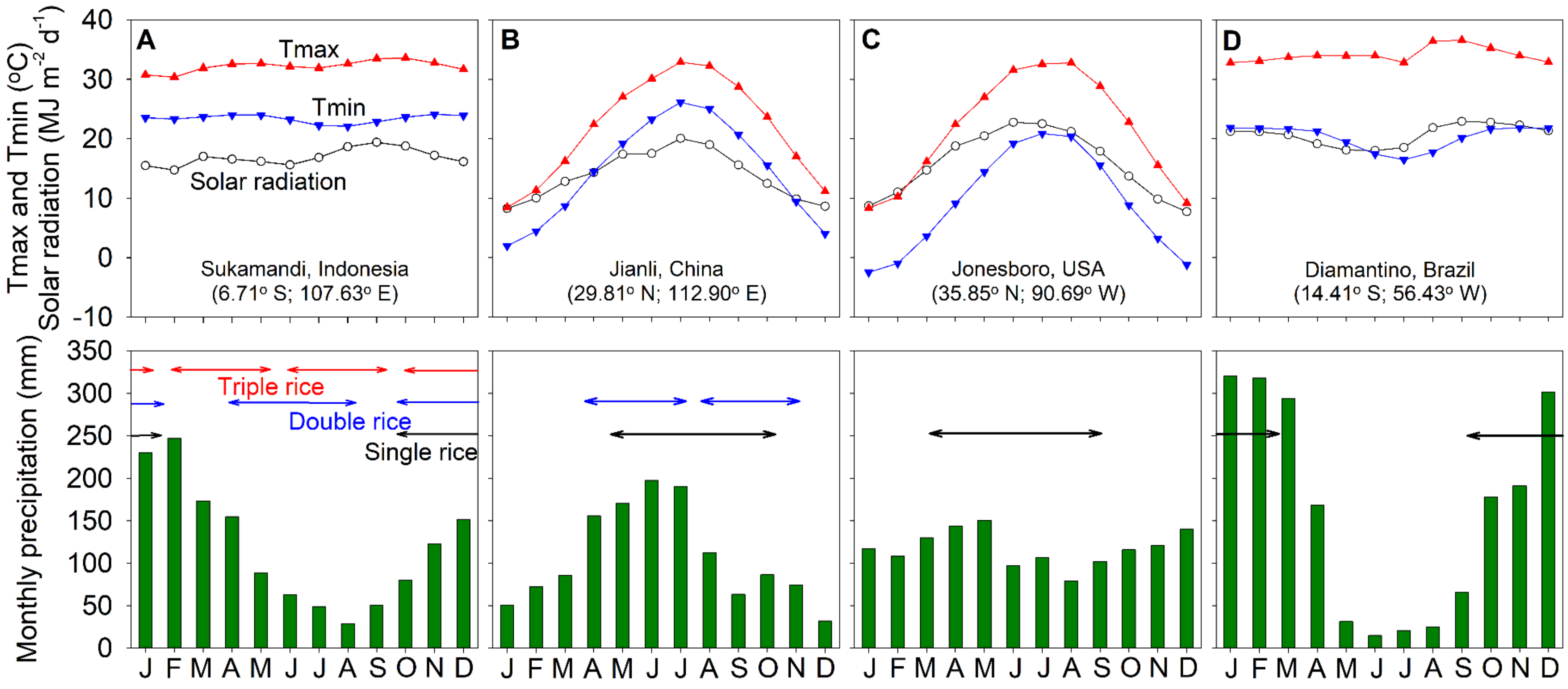

Supplementary Figure 2. Monthly means of solar radiation, maximum (Tmax) and minimum (Tmin) temperatures, and total precipitation. Four sites were selected to illustrate weather patterns for (A) tropical lowland irrigated rice in South-East Asia (Sukamandi, Indonesia), (B) non-tropical lowland irrigated rice in East Asia (Jianli, China), (C) non-tropical lowland irrigated rice in North America (Jonesboro, USA), and (D) tropical rainfed upland rice in South America (Diamantino, Brazil). Arrows indicate the approximate duration of the rice crop cycle(s) at each site. Data are provided in Source Data.

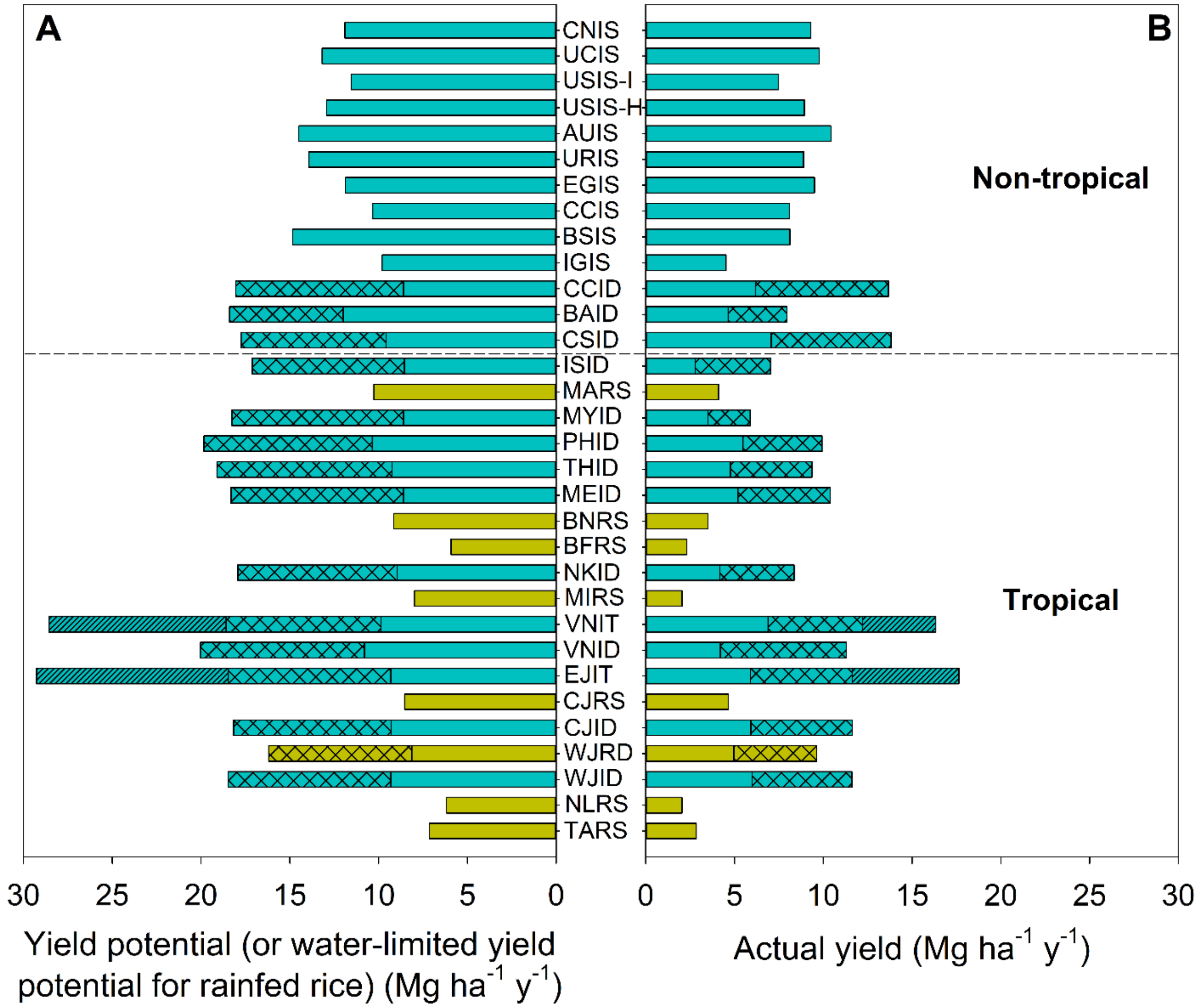

Supplementary Figure 3. Average annual rice yield potential or water-limited yield potential for rainfed rice (left panel) and actual yield (right panel) for each of the 32 rice cropping systems. Cropping systems were sorted according to latitude, from non-tropical (top) to tropical regions (bottom). Blue and brown bars denote irrigated and rainfed systems, respectively. Different bar patterns are used to distinguish yield potential (or water-limited yield potential) for each crop cycle in each cropping system. Cropping system codes are shown in the Supplementary Table 2. Data are provided in Source Data Supplementary Fig. 3.

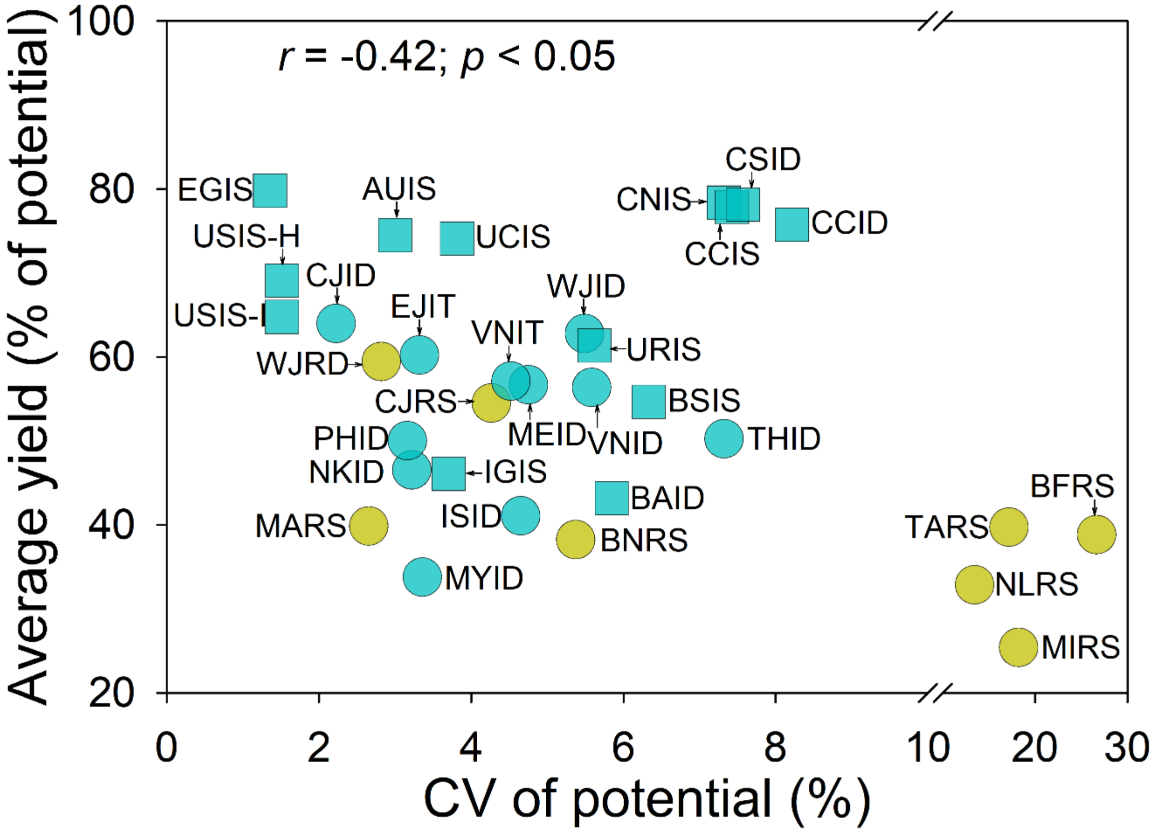

Supplementary Figure 4. Average rice yield, expressed as percentage of yield potential (or water-limited yield potential for rainfed rice) plotted against coefficient of variation (CV) of yield potential across years for each of the 32 rice cropping systems. Symbol type and color are used to distinguish tropical versus non-tropical regions (circles and squares, respectively) and irrigated versus rainfed systems (blue and yellow, respectively). The Pearson's correlation coefficient (*r*) and associated *p*-value are shown (two-tailed Student's *t*-test; n=32 cropping systems). Cropping system codes are shown in Supplementary Table 2. Data are provided in Source Data.

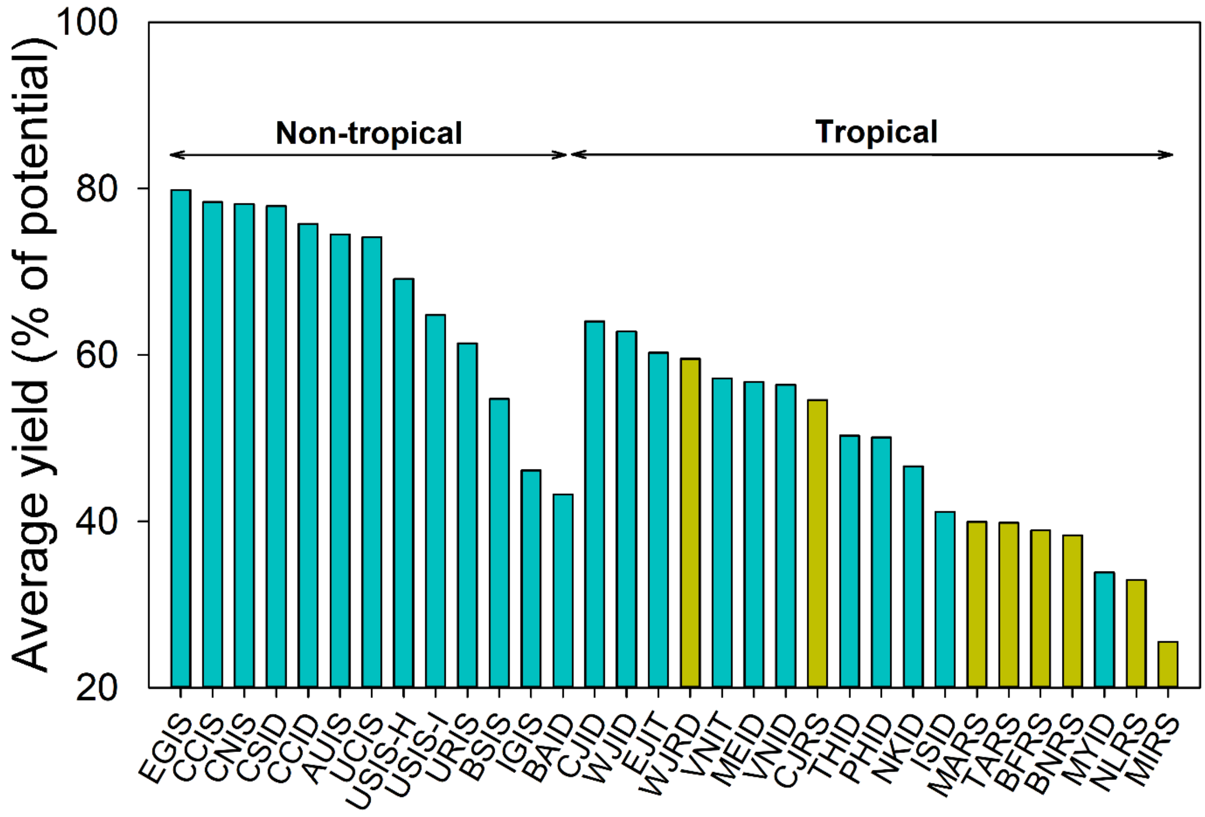

Supplementary Figure 5. Average rice yield, expressed as percentage of yield potential (or water-limited yield potential for rainfed rice) for each of the 32 rice cropping systems, which are grouped into non-tropical and tropical regions, and sort from highest to lowest in each group. Blue and brown bars denote irrigated and rainfed systems. Cropping system codes are shown in Supplementary Table 2. Data are provided in Source Data.

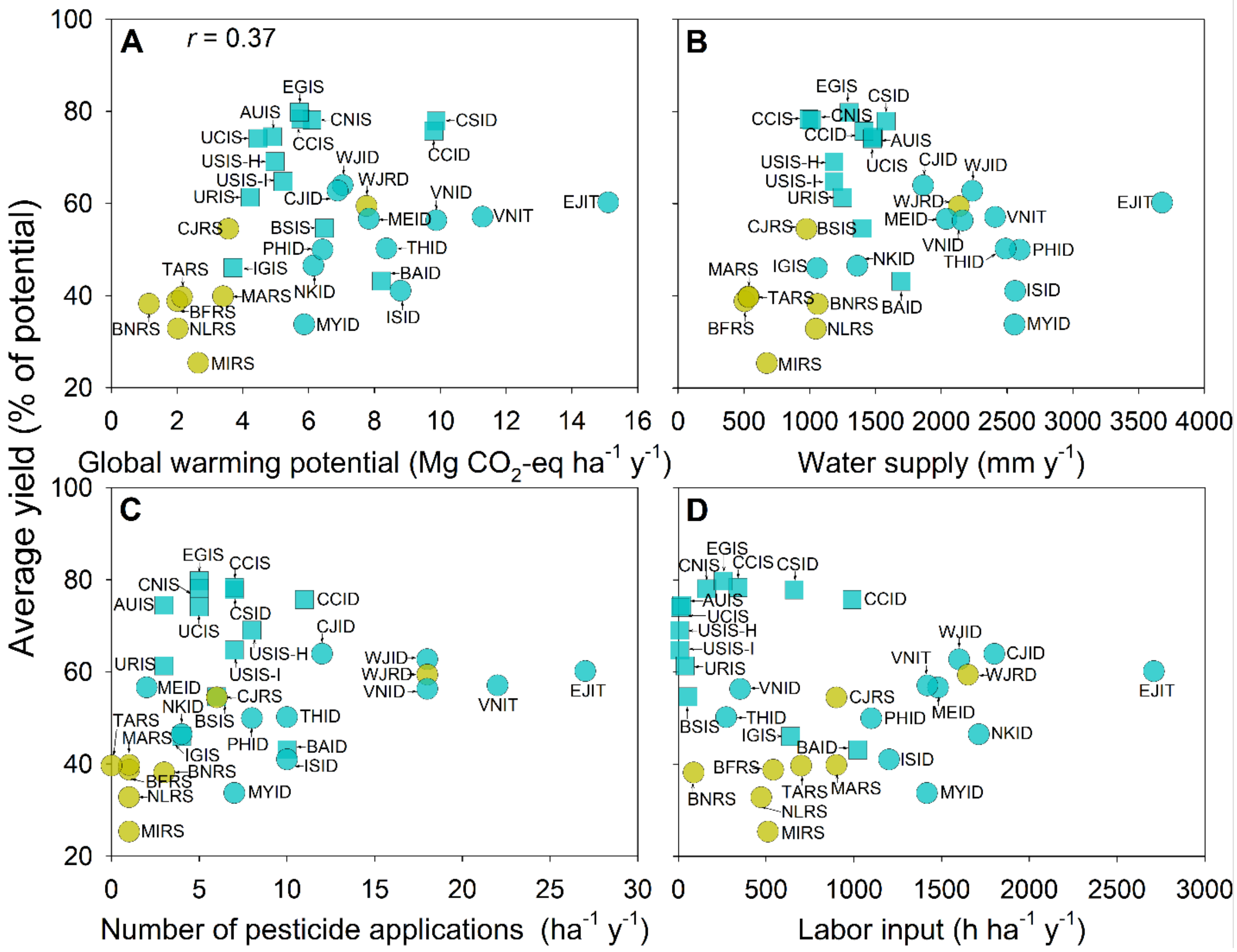

Supplementary Figure 6. Average rice yield, expressed as percentage of yield potential (or water-limited yield potential for rainfed rice) plotted against (A) global warming potential, (B) water supply (irrigation plus in-season precipitation), (C) number of pesticide applications, and (D) labor input per hectare per year. Symbol type and color are used to distinguish tropical versus non-tropical regions (circles and squares, respectively) and irrigated versus rainfed systems (blue and yellow, respectively). Pearson's correlation coefficient (r) is shown only when associations between variables were statistically significant (two-tailed Student's t-test; $p<0.05$; n=32 cropping systems). Statistical analysis is performed using two-tailed Student's t-test (n=32 cropping systems). Cropping system codes are shown in Supplementary Table 2. Data are provided in Source Data.

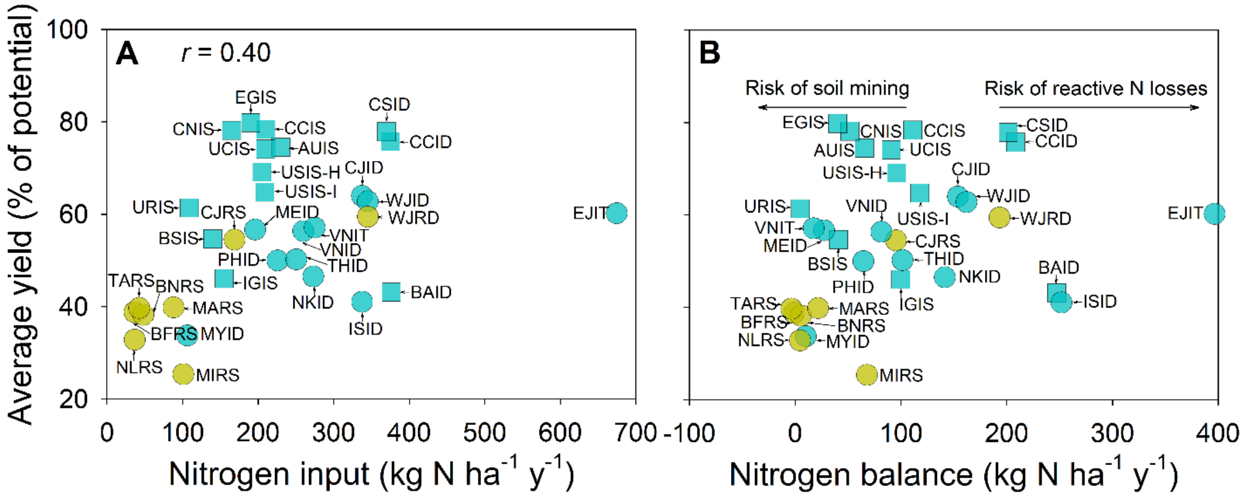

Supplementary Figure 7. Average rice yield, expressed as percentage of yield potential (or water-limited yield potential for rainfed rice) plotted against (A) total nitrogen (N) input (from fertilizer, manure, and fixation) and (B) N balance calculated as external N input minus N removal per hectare per year. Symbol type and color are used to distinguish tropical versus non-tropical regions (circles and squares, respectively) and irrigated versus rainfed systems (blue and yellow, respectively). Pearson's correlation coefficient (*r*) is shown only when associations between variables were statistically significant (two-tailed Student's *t*-test; $p<0.05$; n=32 cropping systems). Cropping system codes are shown in Supplementary Table 2. Data are provided in Source Data.

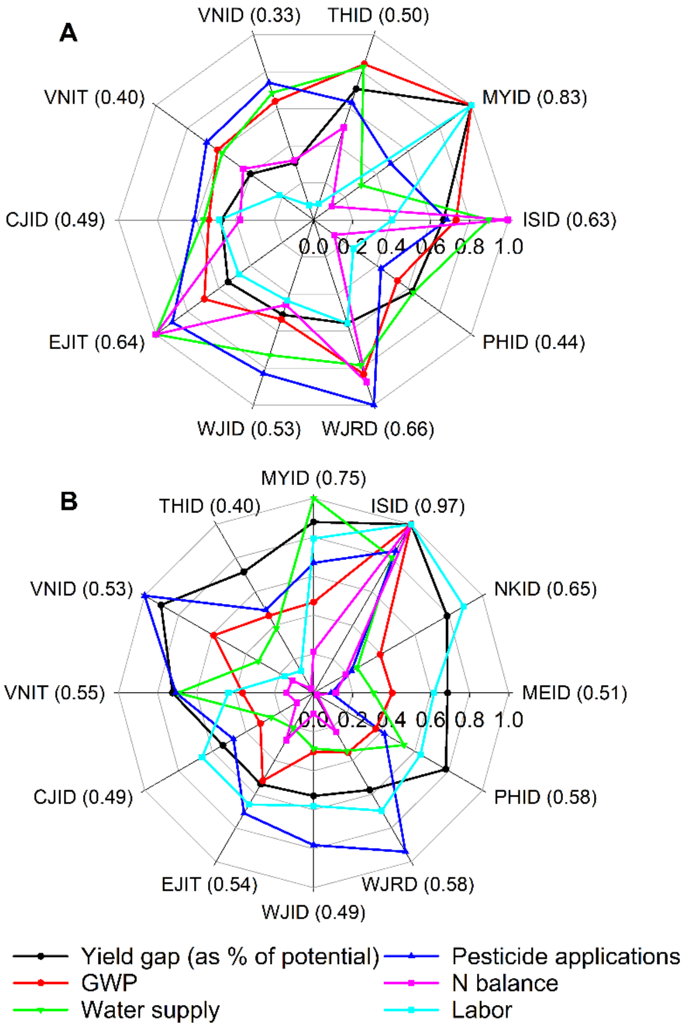

Supplementary Figure 8. Radar chart comparing yield gap (as percentage of yield potential) and yield-scaled metrics including global warming potential (GWP), water supply, number of pesticide applications, nitrogen (N) balance, and labor across 11 rice cropping systems in (A) dry and (B) wet season in tropical region. For each metric, data were normalized relative to the maximum value across all cropping systems, except for the yield-scaled N balance, which was expressed as an absolute deviation from 8 kg N Mg^{-1} grain. Parenthetic values are the performance index of each system, with lower (higher) values indicating better (worse) overall performance. Cropping system codes are shown in Supplementary Table 2. See Methods section for explanation about the calculation of the overall performance index. Cropping systems in Nigeria and Mali are only shown for the wet season data were not available for the dry season. Data are provided in Source Data.

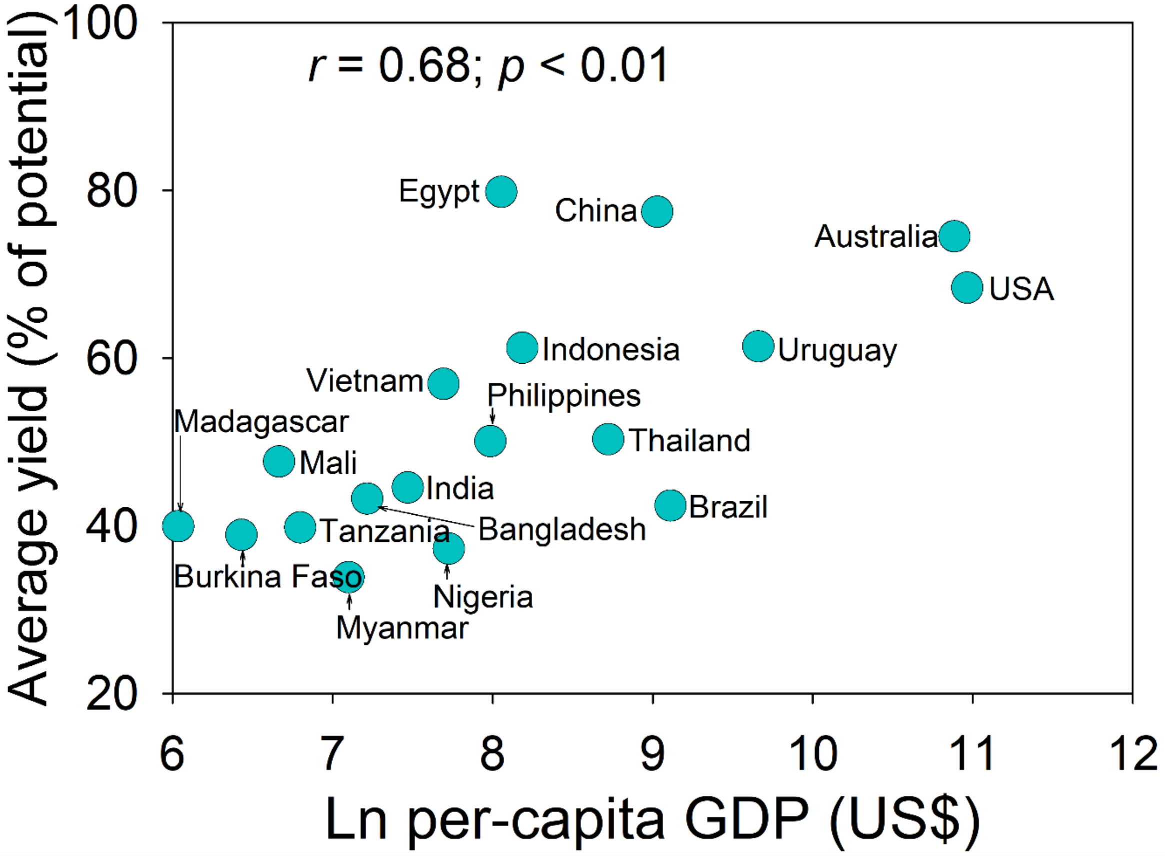

Supplementary Figure 9. Average rice yield, expressed as percentage of yield potential (or water-limited yield potential for rainfed rice) plotted against per-capita gross domestic product for each of the 18 rice-producing countries. Data on per-capita gross domestic product have been log-transformed. Each data point represents an area-weighted value of average rice yield, with the weighting depending upon the rice harvested area of each system in a country. The Pearson's correlation coefficient (*r*) and associated *p*-value are shown (two-tailed Student's *t*-test; *n*=18 countries). Data are provided in Source Data.

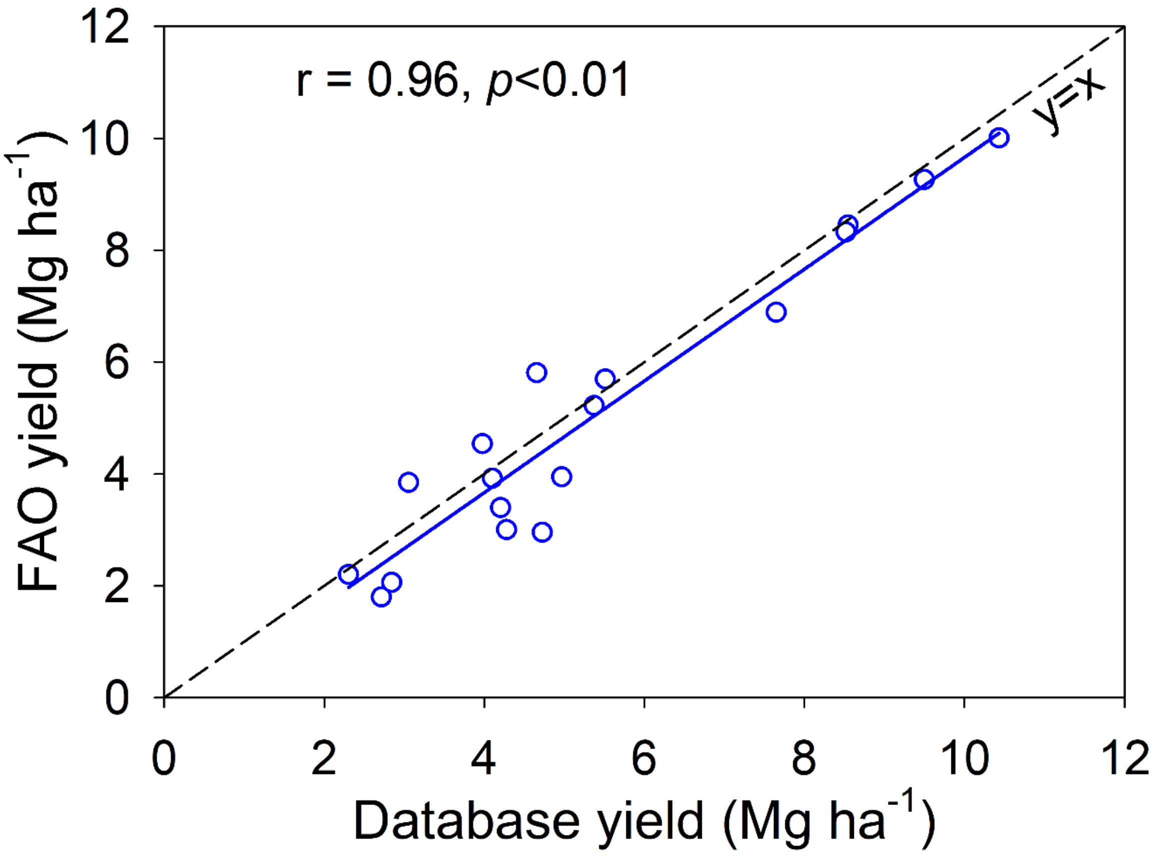

Supplementary Figure 10. National average rice yield reported in FAO versus area-weighted national average yield based on our database actual yields for each of the 18 countries included in the analysis. Dashed diagonal line indicates y = x. Pearson's correlation coefficient (*r*) and associated *p*-value are shown (two-tailed Student's *t*-test; *n*=18 countries). The fitted linear regression model is also shown. Data are provided in Source Data.

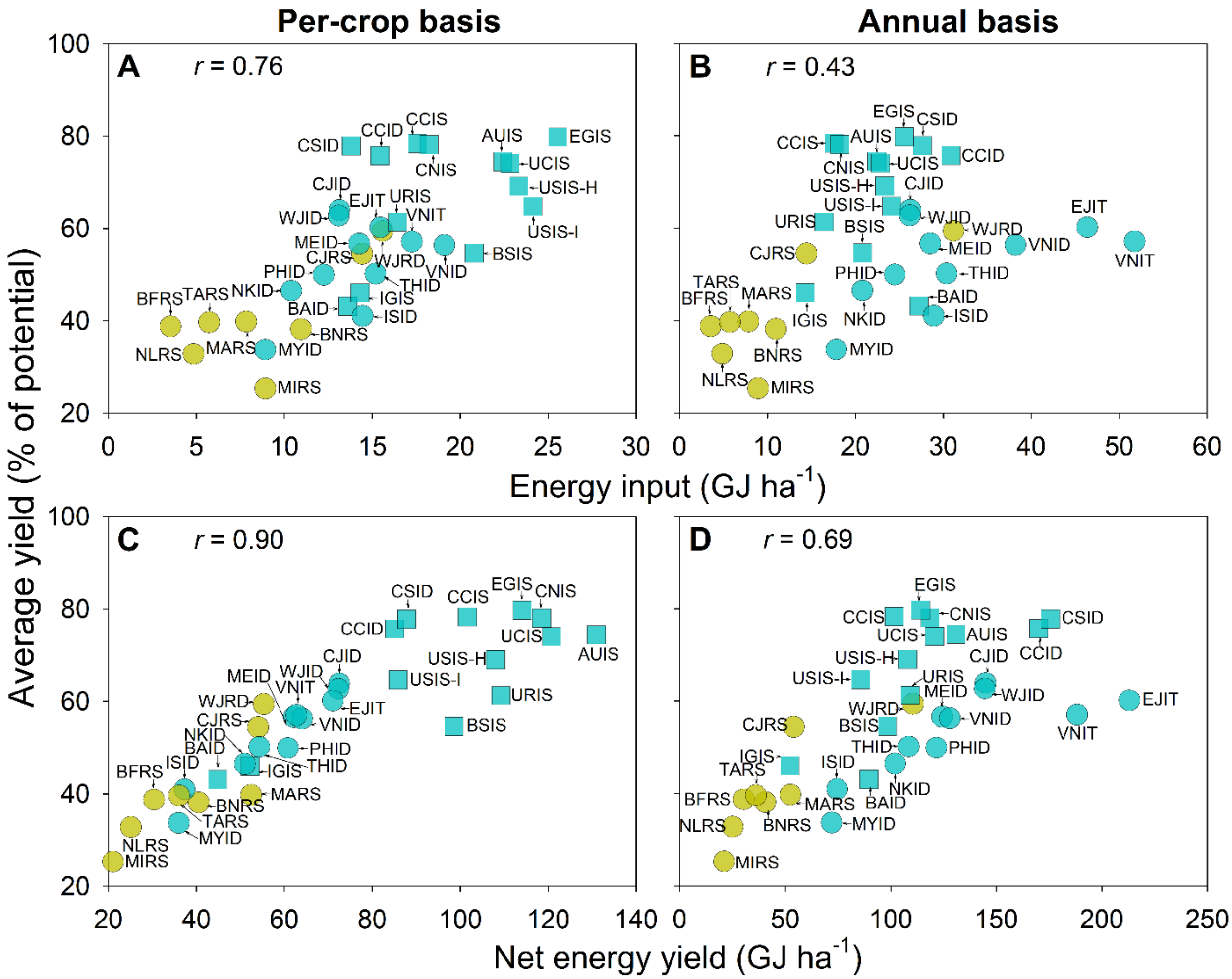

Supplementary Figure 11. Average rice yield, expressed as percentage of yield potential (or water-limited yield potential for rainfed rice) plotted against (A, B) total fossil-fuel energy input (including labor) and (C, D) net energy yield calculated as the difference between energy output and input on a per-crop (A, C) and annual basis (B, D). Symbol type and color are used to distinguish tropical versus non-tropical regions (circles and squares, respectively) and irrigated versus rainfed systems (blue and yellow, respectively). Pearson correlation coefficient (*r*) is shown only when associations between variables were statistically significant (two-tailed Student's *t*-test; $p<0.05$; n=32 cropping systems). Cropping system codes are shown in Supplementary Table 2. Data are provided in Source Data.

Supplementary Tables

Supplementary Table 1. Annual rice harvested area (and percentage of global total), rice production (and percentage of global total), and per-capita gross domestic product (GDP) across 18 countries during the 2015-2017 period. Countries were sorted in a descending order of rice harvested area. Sources: FAO[30]; World Bank[31].

Country	Rice harvested area (kha)	% global rice area	Rice production (MMT)	% global rice production	Per-capita GDP ($\times 10^3$ US$)
India	43456	26	163	22	1.8
China	31030	19	214	28	8.3
Indonesia	15020	9	79	10	3.6
Bangladesh	11218	7	50	7	1.4
Thailand	9891	6	29	4	6.1
Vietnam	7757	5	44	6	2.2
Myanmar	6746	4	26	3	1.2
Philippines	4675	3	18	2	2.9
Nigeria	4547	3	9	1	2.3
Brazil	2030	1	12	2	9.1
Tanzania	1186	1	3	< 1	0.9
USA	1085	1	9	1	57.9
Madagascar	827	1	4	< 1	0.4
Mali	788	< 1	3	< 1	0.8
Egypt	588	< 1	6	1	3.1
Uruguay	162	< 1	1	< 1	15.7
Burkina Faso	159	< 1	< 1	< 1	0.6
Australia	59	< 1	1	< 1	53.4
**Total**		**86**		**88**	

Thousand hectares (kha), million metric tons (MMT).

Supplementary Table 2. Overview of 32 rice cropping systems in 18 countries.

Country	Region	Ecosystem	Water regime	Number of rice cycles†	Dominant plant cultivar	Cropping system code§
Australia (AU)	New South Wales	L	I	S	I	AUIS
Bangladesh (BA)	North	L	I	D	I	BAID
Burkina Faso (BF)	Cascades	L	R	S	I	BFRS
Brazil	North (BN)	U	R	S	I	BNRS
	South (BS)	L	I			BSIS
China	Central (CC)	L	I	S	H	CCIS
				D		CCID
	North (CN)			S	I	CNIS
	South (CS)			D	H	CSID
Egypt (EG)	Delta	L	I	S	I	EGIS
Indonesia	Central Java (CJ)	L	I	D	I	CJID
			R	S		CJRS
	East Java (EJ)		I	T		EJIT
	West Java (WJ)			D		WJID
			R			WJRD
India	Indo-Gangetic Plain (IG)	L	I	S	I	IGIS
	Southern (IS)			D		ISID
Madagascar (MA)	Ambohibary	L	R	S	I	MARS
Mali	Segou (ME)	L	I	D	I	MEID
	Sikasso (MI)		R	S		MIRS
Myanmar (MY)	Ayeyarwady delta	L	I	D	I	MYID
Nigeria	Kano (NK)	L	I	D	I	NKID
	Lafia (NL)		R	S		NLRS
Philippines (PH)	Central Luzon	L	I	D	I	PHID
Thailand (TH)	Central region	L	I	D	I	THID
Tanzania (TA)	Kahama	L	R	S	I	TARS
USA	South (US)	L	I	S	H	USIS-H
					I	USIS-I
	California (UC)					UCIS
Uruguay (UR)	North, Central and East	L	I	S	I	URIS
Vietnam (VN)	Mekong delta	L	I	D	I	VNID
				T		VNIT

Ecosystems: lowland (L); upland (U). Water regimes: irrigated (I) and rainfed (R). Number of rice cycles: single (S), double (D), and triple (T) season rice. Dominant plant cultivar: inbred (I), hybrid (H). † It does not include other crops such as maize, wheat, or soybean (see Supplementary Fig. 1). § Cropping system code consists of country or region ID (first two letters), water regime (third letter), and rice cropping intensity (fourth letter). In the case of the southern USA, hybrid and inbred rice are also distinguished.

Supplementary Table 3. Proportion of rice harvested area in each of selected regions to national total and rice area accounted by each of selected cropping systems to regional total. Cropping system codes are shown in Supplementary Table 2.

Country	Region	Share to national total, %	Cropping system code	Share to regional total, %	Sources
Australia (AU)	New South Wales	85	AUIS	100	[32,33]
Bangladesh (BA)	North	77	BAID	80	[34,35]
Burkina Faso (BF)	Cascades	44	BFRS	75	[32,36]
Brazil	North (BN)	24	BNRS	100	[37,38]
	South (BS)	72	BSIS	100	[32,37]
China	Central (CC)	51	CCIS	50	[39]
			CCID	50	[39]
	North (CN)	19	CNIS	100	[39]
	South (CS)	20	CSID	70	[39]
Egypt (EG)	Delta	100	EGIS	100	[40,41]
Indonesia	Central Java (CJ)	13	CJID	70	[42,43]
			CJRS	20	[42,43]
	East Java (EJ)	15	EJIT	50	[42,43]
	West Java (WJ)	14	WJID	80	[42,43]
			WJRD	10	[42,43]
India	Indo-Gangetic Plain (IG)	50	IGIS	90	[44,45]
	Southern (IS)	30	ISID	70	[44,45]
Madagascar (MA)	Ambohibary	75	MARS	100	[1,36]
Mali	Segou (ME)	64	MEID	60	[1,46]
	Sikasso (MI)	21	MIRS	75	[1,46]
Myanmar (MY)	Ayeyarwady delta	63	MYID	50	[43,47]
Nigeria	Kano (NK)	21	NKID	90	[1,36]
	Lafia (NL)	68	NLRS	60	[1,36]
Philippines (PH)	Central Luzon	55	PHID	85	[43,48]
Thailand (TH)	Central region	15[§]	THID	65	[43,49,50]
Tanzania (TA)	Kahama	66	TARS	100	[1,36]
USA	South (US)	80	USIS-H	50	[32,51]
			USIS-I	50	[32,51]
	California (UC)	20	UCIS	100	[32,51]
Uruguay (UR)	North, Central and East	100	URIS	100	[32,52]
Vietnam (VN)	Mekong delta	54	VNID	30	[53,54]
			VNIT	60	[53,54]

[§] Central region accounts for nearly half of national total irrigated rice area in Thailand[50].

Supplementary Table 4. Key information on agronomic and labor inputs and grain yield in each rice cropping system. Values on applied inputs and yields are expressed per hectare on a per-crop basis. Cropping system codes are shown in Supplementary Table 2.

Cropping system code	SR (kg)	Fertilizer (kg)			Manure		Pesticide		Irrigation (mm)	Labor (h)	Yield (Mg)
		N	P	K	Rate	Type	No.	Rate			
AUIS	150	200	25	0	0		3	2.6	1250 (0)	19	10.4
BAID	9	158	60	57	175	P	5	4.1	350 (0)	512	4.0
BFRS	65	6	4	4	19	A	1	0.2	0 (0)	540	2.3
BNRS	70	45	33	62	0		3	2	0 (0)	86	3.5
BSIS	90	110	58	62	0		6	4.6	900 (100%)	55	8.1
CCIS	21	180	70	100	0		7	4.8	500 (100%)	340	8.1
CCID	23	158	59	90	0		6	3.5	240 (100%)	495	6.8
CNIS	75	135	55	75	0		5	3.5	700 (100%)	160	9.3
CSID	30	155	17	94	0		4	1.5	299 (100%)	330	6.9
EGIS	100	160	38	57	0		5	3.9	1300 (100%)	256	9.5
CJID	20	135	50	35	600	A	6	4.3	184 (0)	900	5.8
CJRS	20	135	50	35	600	A	6	4.3	174 (100%)	900	4.7
EJIT	30	192	30	35	500	A	9	3.3	798 (20%)	903	5.9
WJID	27	143	34	44	0		9	6.1	777 (0)	800	5.8
WJRD	27	143	34	44	0		9	6.1	725 (50%)	825	4.8
IGIS	75	125	30	30	0		4	2.3	307 (100%)	640	4.5
ISID	70	135	45	45	1250	P	5	2.2	620 (0)	600	3.5
MARS	107	28	0	0	5000	A	1	0.1	0 (0)	900	4.1
MEID	50	68	19	4	72	A	1	0.2	750 (100%)	740	5.2
MIRS	76	71	11	10	14	P	1	0.1	0 (0)	510	2.0
MYID	123	23	1	0	0		4	2.9	408 (0)	694	3.1
NKID	35	107	16	31	0		2	0.2	550 (0)	856	4.2
NLRS	117	7	0	1	0		1	0.2	0 (0)	472	2.0
PHID	86	83	36	28	0		4	3.2	565 (30%)	550	5.0
THID	125	95	38	21	113	A	5	4.2	642 (50%)	140	4.7
TARS	62	12	1	0	40	A	0	0	0 (0)	700	2.8
USIS-H	25	175	20	56	0		8	3.5	720 (100%)	7	8.9
USIS-I	67	179	20	56	0		7	3.5	720 (100%)	7	7.5
UCIS	170	180	22	29	0		5	6.3	1450 (0)	11	9.8
URIS	147	79	17	28	0		3	2.3	690 (50%)	38	8.5
VNID	101	100	65	61	0		9	6.9	718 (100%)	176	5.7
VNIT	204	62	26	21	0		7	5.6	478 (70%)	473	5.4

SR: seeding rate; synthetic fertilizer nutrient application: nitrogen (N), phosphorus (P), and potassium (K) expressed as elemental nutrient; other nutrient application: animal manure (A) or plant compost (P) and associated rates in fresh weight (kg ha^{-1}); number of pesticide applications: number and total amount (kg a.i. ha^{-1}), irrigation water amount (and percentage of pumping for irrigation), labor input, and grain yield at 14% MC.

Supplementary Table 5. Key information on crop management in each rice cropping system. Cropping system codes are shown in Supplementary Table 2. See footnote for abbreviations.

Cropping system code	Tillage method	Establishment method	Mechanization level	Straw management	Weed control	Field size (ha)
AUIS	F	D	H	B	C	60
BAID	M	T	I	R	C	0.2
BFRS	F	D	L	R	C	0.2
BNRS	F	D	H	L	C	75
BSIS						114
CCIS	F	T	I	L	C	0.3
CCID						0.1
CNIS			H			0.9
CSID			I			0.1
EGIS	F	D	I	B	C	0.5
CJID	F	T	L	B	C+M	0.2
CJRS						0.2
EJIT						1
WJID						1
WJRD						1
IGIS	F	T	I	L	C	1.2
ISID						1.2
MARS	F	T	L	R	M	0.1
MEID	F	T	L	R	C+M	1.3
MIRS		D				0.4
MYID	F	T	I	B	M	2.5
NKID	F	T	L	B	C+M	0.5
NLRS		D				0.3
PHID	M	T	I	R	C+M	1.1
THID	F	D	I	B	C+M	2.8
TARS	F	T	L	R	M	1.5
USIS-H	F	D	H	L	C	84
USIS-I						84
UCIS						40
URIS	M	D	H	L	C	90
VNID	M	D	I	B	C+M	2.1
VNIT						2.6

Tillage method: full (F), minimum (M); crop establishment method: direct seeded (D), transplanted (T); Mechanization level: high (H), intermediate (I), low (L); straw management: left in field (L), burn (B), remove out of the field (R); weed control: manual (M), chemical (C).

Supplementary Table 6. Questionnaire used in our study to collect yield and management data for each of the 32 cropping systems.

No.	Variable			
1	Country			
2	Region			
3	Ecosystem (upland/lowland)			
4	Water regime (irrigated/rainfed)			
5	Cropping system			
6	Annual crop calendar	Cycle 1	Cycle 2	Cycle 3
	Establishment			
	Harvest			
	**DOMINANT** PRACTICES PER CYCLE	Source(s) of management data:		
7	Crop establishment method			
8	Field size (ha per field)			
9	Is this operation mechanized? (yes/no)			
	Tillage			
	Puddling			
	Sowing or transplanting			
	Fertilizing			
	Spraying			
	Weeding			
	Harvesting			
	Grain drying			
10	Labor requirements (hour-person per cycle ha^{-1})			
11	Straw management			
12	Tillage method			
13	Seeding rate (kg ha^{-1})			
14	Total N rate (kg ha^{-1})			
15	Total P rate (kg ha^{-1})			
16	Total K rate (kg ha^{-1})			
17	Other nutrients (kg ha^{-1}), if any			
18	Manure source & rate (kg ha^{-1}) -cow, goat, poultry, compost			
19	Lime (kg ha^{-1})			
20	Total irrigation amount (indicate m^3 ha^{-1} or mm depth per ha)			
21	Source and type of irrigation			
22	Source of energy for pumping			
23	Names of the most commonly used insecticides			
24	Names of the most commonly used fungicides			
25	Names of the most commonly used herbicides			
26	Total active ingredient of insecticides (kg ha^{-1}) & No. of spraying times [X]			
27	Total active ingredient of fungicides (kg ha^{-1}) & No. of spraying times [X]			
28	Total active ingredient of herbicides (kg ha^{-1}) & No. of spraying times [X]			

29	Other chemicals? (e.g., rodenticide, molluscicide, nematicide, growth regulators/hormones) -Indicate product (or active ingredient) and rate	
30	YEAR-SPECIFIC YIELD DATA	Source(s) of yield data:
		Cycle 1 Cycle 2 Cycle 3
	Paddy yield at 14% MC (kg ha^{-1}) -year: xxxx	
	Paddy yield at 14% MC (kg ha^{-1}) -year: xxxx	
	Paddy yield at 14% MC (kg ha^{-1}) -year: xxxx	

Supplementary Table 7. Sources of data on crop management practices, applied inputs, and actual yield, and yield potential and yield potential (or water-limited yield potential for rainfed rice). Cropping system codes are shown in Supplementary Table 2.

Cropping system code	Crop management practices, applied inputs, and actual yield	Yield potential
AUIS	Rice Marketing Board Annual Report	Lacy et al.[2]
BAID	Metrics and Indicators for Tracking in GRiSP (MISTIG) survey, Bureau of Statistics	GYGA
BFRS	Yield Gap Survey	GYGA
BNRS<br>BSIS	Field survey	GYGA
CCIS<br>CCID<br>CNIS<br>CSID	Field survey	GYGA
EGIS	National Rice Campaign, Expert opinion	GYGA
CJID<br>CJRS<br>EJIT<br>WJID<br>WJRD	Field survey, The Office of Agriculture at Grobogan Regency<br><br>Field survey, Agricultural services<br>Field survey, Statistics Indonesia	GYGA
IGIS<br>ISID	Crop Production Statistics Information System, India	GYGA
MARS	Field survey	GYGA
MEID<br>MIRS	Yield Gap Survey	GYGA
MYID	MISTIG survey, Metrics and Indicators for Tracking in RICE CRP (MISTIR) survey	GYGA
NKID<br>NLRS	Field survey, Expert opinion<br>Yield Gap Survey	GYGA
PHID	MISTIG and MISTIR survey, Philippine Statistics Authority	GYGA
THID	Closing Rice Yield Gaps in Asia (CORIGAP) survey	GYGA
TARS	Yield Gap Survey	GYGA
USIS-H<br>USIS-I<br>UCIS	United States Department of Agriculture (USDA)<br><br>UC Cooperative Extension, USDA	GYGA
URIS	Agricultural statistical yearbook (DIEA, MGAP), Summary of Rice seasons data base reports, Rice working group (INIA), Scientific and local publications, Expert's opinion	GYGA
VNID<br>VNIT	MISTIG and MISTIR survey, General Statistics Office	GYGA

GYGA: Global Yield Gap Atlas (www.yieldgap.org)

Supplementary Table 8. Cross-validation of average farmer yield (Mg ha^{-1}), nitrogen (N) fertilizer rate (kg N ha^{-1}), labor (h), and irrigation (mm) per hectare per crop estimated in our study (S) with those reported in the literature (L) for 10 selected countries for which data are available.

	Yield		N rate		Labor		Irrigation		Sources
	S†	L§	S†	L§	S†	L§	S†	L§	
USA	7.5-9.8	7.4-9.8	175-180	167-234	7-11	8-11	CA: 1450	1422-1524	51,55-61
							S: 720	762-838	
Uruguay	8.6	8.4-8.5	79	71-80	38	34	690	789-804	52,62-66
South Brazil	8.1	8.6-8.8	110	117	55	80	900	1130-1150	67-70
India	3.5-4.5	2.4-5.5	125-135	106-151	600-640	800	307-620	344-1188	71-76
China	6.8-9.3	5.5-9.4	135-180	128-209	160-495	293-768	250-700	270-825	77-85
Myanmar	3.1	2.3-3.4	23	18-48	694	544-710		n.a.	86-90
Thailand	4.7	4.8-5.3	95	97-106	140	108-144	D: 784	D: 532-1187	72,86,89,91-95
							W: 594	W: 471-970	
Vietnam	5.4-5.7	4.7-6.4	62-100	87-111	176-473	176-636	D: 880	D: 321-1075	54,72,86,87,89,96,97
							W: 555	W: 465-700	
Indonesia	4.7-5.9	4.3-5.9	135-192	106-235	800-903	840-1576		n.a.	86,87,89,98,99
Philippines	5.0	4.2-4.9	83	107-114	550	420-512	D: 750	D: 427-946	89,100-106
							W: 380	W: 96-482	

† Values indicate the range of averages provided for each cropping system per country, disaggregated in the case of irrigation based on crop season (D: dry; W: wet) for tropical countries and also region (CA: California; S: South) for USA.

§ Data on yield, N rate, and labor from previous studies based on on-farm survey; irrigation data from previous studies measured in experimental treatments that followed farmers' practice (on-farm data on irrigation were not available). Values indicate the range of averages reported for each variable across studies. Only studies published after year 2000 were included in the cross-validation.

n.a.: not available.

Supplementary Table 9. Means of daily solar radiation and maximum (Tmax) and minimum (Tmin) temperature, and total rainfall during the rice growing season based on at least five years of measured weather data at each site. Values are averages across crop cycles in the case of systems with more than one cycle. Cropping system codes are shown in Supplementary Table 2.

Cropping system code	Weather station	Latitude (degree)	Longitude (degree)	Radiation (MJ m^{-2} d^{-1})	Tmax (°C)	Tmin (°C)	Total rainfall (mm)
AUIS	Wagga Wagga	-35.11	147.36	24.1	28.8	13.7	232
BAID	Dhaka	23.81	90.41	17.5	31.7	22.8	498
BFRS	Fada Ngourma	12.06	0.37	20.1	33.5	22.6	494
BNRS	Diamantino	-14.41	-56.43	18.1	28.0	18.1	1061
BSIS	Santa Maria	-29.69	-53.81	23.2	25.4	15.9	501
CCIS	Jianli	29.81	112.90	16.9	29.5	22.1	495
CCID	Yongzhou	26.42	111.61	18.0	29.2	21.6	457
CNIS	Fujin	47.25	132.04	18.1	23.7	13.2	316
CSID	Gaoyao	23.03	112.46	17.9	30.1	23.1	492
EGIS	Cairo	30.04	31.24	26.6	34.0	20.7	1
CJID	Blora	-7.01	111.38	17.9	28.0	22.8	747
CJRS				19.1	28.7	22.9	802
EJIT	Perak II	-7.21	112.73	18.3	33.6	24.9	427
WJID	Sukamandi	-6.71	107.63	16.6	32.2	23.6	341
WJRD							
IGIS	Modipuram	29.07	77.71	16.0	29.8	17.4	749
ISID	Bhubaneshwar	20.30	85.82	17.0	33.0	21.9	661
MARS	Ambohibary	-19.62	47.13	20.8	21.3	12.4	534
MEID	Segou	13.43	-6.25	22.0	35.8	24.5	270
MIRS	Sikasso	11.32	-5.70	19.4	30.4	21.8	675
MYID	Bago	17.32	96.47	18.6	33.7	23.0	870
NKID	Kano	12.00	8.59	22.0	34.0	19.1	131
NLRS	Enugu	6.46	7.55	15.4	31.4	22.6	1046
PHID	Dagupan	16.09	120.35	19.1	32.2	23.3	734
THID	Nakhon Sawan	15.7	100.12	19.2	34.7	24.2	603
TARS	Mwanza	-2.52	32.92	19.8	28.2	17.6	535
USIS-H	Jonesboro	35.85	-90.69	21.1	29.3	16.8	465
USIS-I							
UCIS	Colusa	39.21	-122.01	23.8	30.5	12.5	27
URIS	Treinta y Tres	33.23	-54.36	21.7	26.7	14.6	560
VNID	Can Tho	10.05	105.75	19.8	32.3	24.7	364
VNIT				19.7	32.2	24.8	325

Supplementary Table 10. Emission factors used for estimating greenhouse gases emissions from manufacturing, packaging, and transportation of agricultural inputs.

Inputs	Unit	Emission factor (CO_2-eq kg unit^{-1})	References
Machinery	MJ	0.071	Dyer and Desjardins[107]; Khoshnevisan et al.[108]
Diesel	l	2.76	Khoshnevisan et al.[108]; Pishgar-Komleh et al.[109]
LPG	l	1.50	EPA[17]
Electricity	kWh	0.78	Casey and Holden[110]; Mondani et al.[111]
Nitrogen (N)	kg	4.77	Lal[112]; Pathak and Wassman[113]
Phosphorus (P_2O5)	kg	0.73	Lal[112]; Pathak and Wassman[113]
Potassium (K_2O)	kg	0.55	Lal[112]; Pathak and Wassman[113]
Manure	kg	0.13	Li and Kotelko[114]; Mondani et al.[111]
Pesticides	kg a.i. §	18.7	Lal[112]; Yang et al.[115]
Seed	kg	0.78	Guo et al.[116]

§ a.i., active ingredient.

Supplementary Table 11. Fuel consumption and operation time of machine in different field operations in rice production.

Field operation	Fuel consumption ($l\ ha^{-1}$)	Operation time ($h\ ha^{-1}$)	References
Land preparation (full tillage)	38	5.0	Hokazono and Hayashi[117]; Sims et al.[16]
Land preparation (minimum tillage)	23	3.5	Hokazono and Hayashi[117]; Sims et al.[16]
Sowing	5	0.2	Fusi et al.[118]; Sims et al.[16]
Transplanting	15	3.0	Harada et al.[119]
Fertilizing	2.5	0.25	Fusi et al.[118]
Spraying	1.5	0.2	Fusi et al.[118]; Sims et al.[16]
Weeding	1.5	0.2	Fusi et al.[118]; Sims et al.[16]
Harvesting	10	1.0	Fusi et al.[118]; Sims et al.[16]

Supplementary Table 12. Embodied energy of agricultural inputs and output.

Inputs	Unit	Energy equivalent (MJ unit^{-1})	References
Machinery	kg*y	12.5	Pellegrini and Fernández[11]; Mikkola and Ahokas[120]
Diesel	l	45.5	Alluvione et al.[121]; Arizpe et al.[122]
Nitrogen (N)	kg	45.5	Pellegrini and Fernández[11]; IEA[123]
Phosphorus (P_2O_5)	kg	14.2	Pellegrini and Fernández[11]; Meul et al.[124]
Potassium (K_2O)	kg	10.0	Pellegrini and Fernández[11]
Manure	kg	0.3	Wu et al.[125]; Mondani et al.[111]
Pesticides	kg a.i. †	120	Lal et al.[126]
Seed §	kg	30.6	West and Marland[127]; EIA[128]
Electricity	kWh	3.6	Zhang et al.[129]
Labor	h	1.96	Singh et al.[130]
Rice grain	kg	14.7	Lal et al.[126]

† a.i., active ingredient

§ Using dollar to energy conversion of 5.45 MJ US $$^{-1}$ (an average from 2015-2017) for rice seed[128] following West and Marland[127]. Seed prices are from Xie and Hardy[131] and Peng[132].

Supplementary Table 13. The assumed fraction of the total amount of straw remaining in the field and the assumed fraction of nitrogen nutrient lost from crop residues by burning or leaching under different straw managements.

Straw management	Straw remaining, %	Nitrogen lost, %
Left in field	90	20
Burn	90	90
Remove out of field	10	90

Data from Dobermann et al[21].

Supplementary Table 14. Pearson's correlation coefficients among average yield (% of potential), resource inputs, resource-use efficiency, and environmental impact parameters. Metrics are shown on area and yield-scaled basis and computed based on average across the rice crop cycles within each cropping system.

Metric	Yield (% Yp)	On area basis (per ha)							On yield-scaled basis (per Mg)				
		GWP	Water	Pest	N	Labor	Energy	NB	GWP	Water	Pest	Labor	NB
GWP	0.76***												
Water	0.34*	0.44**											
Pest	0.51***	0.55***	0.40*										
N	0.75***	0.75***	0.41	0.64***									
Labor	-0.37**	-0.41**	-0.48***	-0.09	-0.19								
Energy	0.76***	0.81***	0.65***	0.60***	0.75***	-0.60***							
NB	0.39**	0.48***	0.17	0.57***	0.85***	0.07	0.41**						
YGWP	-0.60***	-0.12	-0.28	-0.15	-0.21	0.37**	-0.42**	0.15					
YWater	-0.72***	-0.55***	0.14	-0.27	-0.54***	0.20	-0.52***	-0.27	0.53***				
YPest	0.01	0.13	0.25	0.82***	0.29	0.23	0.17	0.45**	0.21	0.19			
YLabor	-0.72***	-0.67***	-0.58***	-0.43**	0.52***	0.83***	-0.83***	-0.17	0.61***	0.51***	-0.00		
YNB	-0.10	0.14	-0.03	0.28	0.50***	0.28	0.06	0.82***	0.55***	0.07	0.44**	0.20	
NEY	0.90***	0.77***	0.51***	0.36**	0.67***	-0.58***	0.82***	0.24	-0.65***	-0.67***	-0.18	-0.81***	-0.22

Asterisks indicate significance based on Student's *t*-test *p*-value at * $p<0.1$, ** $p<0.05$, *** $p<0.01$. Variables: average yield expressed as % of potential (Yp); GWP: global warming potential (Mg CO_2-eq); water supply, which is the sum of irrigation and in-season precipitation (Water, mm); number of pesticide applications (Pest, unitless); nitrogen (N) input and balance (NB, kg N); labor (h); energy input (Energy) and net energy yield (NEY) (GJ); yield-scaled GWP (YGWP, Mg CO_2-eq Mg^{-1} grain); yield-scaled water supply (YWater, mm Mg^{-1} grain); yield-scaled number of pesticide applications (YPest, No. Mg^{-1} grain); yield-scaled labor (YLabor, h Mg^{-1} grain); yield-scaled nitrogen balance (YNB, kg N Mg^{-1} grain).

Supplementary References

1. Global Yield Gap Atlas - GYGA. (accessed 5 July 2019); www.yieldgap.org/home
2. Lacy, J., Clampett, W. & Nagy, J. *Bridging the rice yield gap in Australia. Bridging the rice yield gap in the Asia-Pacific region* (Food and Agriculture Regional Office for Asia and the Pacific, Bangkok, Thailand, 2000).
3. Bouman, B. A. M., Kropff, M. J., Tuong, T. P., Wopereis, M. C. S., ten Berge, H. F. M. & van Laar, H. H. *ORYZA2000: modeling lowland rice* (International Rice Research Institute and Wageningen University and Research Centre, Los Baños, Philippines and Wageningen, Netherlands, 2001).
4. Yuan, S., Peng, S. & Li, T. Evaluation and application of the ORYZA rice model under different crop managements with high-yielding rice cultivars in central China. *Field Crops Res.* **212**, 115-125 (2017).
5. IPCC. *IPCC 2019 Refinement to the 2006 IPCC guidelines for national greenhouse gas inventories* (Switzerland, 2019).
6. van Groenigen, J. W., Velthof, G., Oenema, O., van Groenigen, K. J. & van Kessel, C. Towards an agronomic assessment of N_2O emissions: a case study for arable crops. *Eur. J. Soil Sci.* **61,** 903-913 (2010).
7. IPCC. *2006 IPCC guidelines for national greenhouse gas inventories* (Institute for Global Environmental Strategies, Hayama, Kanagawa, Japan, 2006).
8. IPCC. *Climate change 2007: The Physical Science Basis. Contribution of Working Group I to the Fourth Assessment Report of the Intergovernmental Panel on Climate Change* (Cambridge University Press, Cambridge, United Kingdom and New York, USA, 2007).
9. Mosier, A. R., Halvorson, A. D., Reule, C. A. & Liu, X. J. Net global warming potential and greenhouse gas intensity in irrigated cropping systems in northeastern Colorado. *J. Environ. Qual.* **35,** 1584-1598 (2006).
10. Stout, B. A. *Handbook of energy for world agriculture* (Elsevier, New York, USA, 1991).
11. Pellegrini, P. & Fernández, R. J. Crop intensification, land use, and on-farm energy-use efficiency during the worldwide spread of the green revolution. *Proc. Natl Acad. Sci. USA* **115,** 2335-2340 (2018).
12. Daccache, A., Ciurana, J. S., Diaz, J. R. & Knox, J. W. Water and energy footprint of irrigated agriculture in the Mediterranean region. *Environ. Res. Lett.* **9,** 124014 (2014).

13. Keller, J. & Bliesner, R. B. *Sprinkler and Trickle Irrigation* (Chapman and Hall, New York, USA, 1990).
14. Billiris, M. A. *Measuring the Energy Required to Dry Rice in Commercial Rice Dryers, Theses and Dissertations* (University of Arkansas, Fayetteville, Arkansas, USA, 2013).
15. Jittanit, W., Saeteaw, N. & Charoenchaisri, A. Industrial paddy drying and energy saving options. *J. Stored Prod. Res.* **46,** 209-213 (2010).
16. Sims, R., Flammini, A., Puri, M. & Bracco, S. *Opportunities for agri-food chains to become energy-smart* (UN Food and Agricultural Organization, Rome and GIZ, 2015).
17. EPA. *Emission Factors for Greenhouse Gas Inventories* (US Environmental Protection Agency, Washington DC, USA, 2014).
18. Grassini, P. & Cassman, K. G. High-yield maize with large net energy yield and small global warming intensity. *Proc. Natl Acad. Sci. USA* **109,** 1074-1079 (2012).
19. Dobermann, A. & Witt, C. *The potential impact of crop intensification on carbon and nitrogen cycling in intensive rice systems. Carbon and nitrogen dynamics in flooded soils* (International Rice Research Institute, Los Baños, Philippines, 2000).
20. Connor, D. J., Loomis, R. S. & Cassman, K. G. *Crop ecology: productivity and management in agricultural systems* (Cambridge Univ Press, Cambridge, United Kingdom, 2011).
21. Dobermann, A., Witt, C. & Dawe, D. *Increasing productivity of intensive rice systems through site-specific nutrient management* (Science Publishers Inc. and International Rice Research Institute, Enfield NH, USA and Los Baños, Philippines, 2004).
22. Herridge, D. F., Peoples, M. B. & Boddey, R. M. Global inputs of biological nitrogen fixation in agricultural systems. *Plant Soil* **311,** 1-18 (2008).
23. Lassaletta, L., Billen, G., Grizzetti, B., Anglade, J. & Garnier, J. 50 year trends in nitrogen use efficiency of world cropping systems: the relationship between yield and nitrogen input to cropland. *Environ. Res. Lett.* **9,** 105011 (2014).
24. Yoshida, T. & Ancajas, R. R. Nitrogen fixing activity in upland and flooded rice fields. *Soil Sci. Soc. Am. Proc.* **37,** 45-46 (1973).
25. Dobermann, A. & Fairhurst, T. H. *Nutrient disorders and nutrient management* (Potash and Phosphate Institute, Potash and Phosphate Institute of Canada and International Rice Research Institute, Singapore, 2000).

26. Khush, G. Productivity improvements in rice. *Nutr. Rev.* **61,** S114-116 (2003).
27. Kovach, J., Petzoldt, C., Degni, J. & Tette, J. A method to measure the environmental impact of pesticides. *NY Food Life Sci. Bull.* **139,** 1-8 (1992).
28. Pandey, S. et al. *Rice in the global economy: strategic research and policy issues for food security* (International Rice Research Institute, Los Baños, Philippines, 2010).
29. Kumar, V. & Ladha, J. K. Direct seeding of rice: recent developments and future research needs. *Adv. Agron.* **111,** 297-413 (2011).
30. FAO. FAOSTAT production data. (accessed 5 July 2019); www.fao.org/faostat/en/#data.
31. World Bank. DataBank. (accessed 5 July 2019); www.databank.worldbank.org/home.aspx.
32. GRiSP (Global Rice Science Partnership). *Rice almanac, 4th edition* (International Rice Research Institute, Los Baños, Philippines, 2013).
33. Australian Bureau of Statistics. Agricultural Commodities. (accessed 2 August 2021); http://www.abs.gov.au/statistics/industry/agriculture.
34. Bangladesh Bureau of Statistics. Yearbook of Agricultural Statistics-2018. (accessed 2 August 2021); http://www.bbs.gov.bd.
35. Islam, A. B. M. J., Shahidullah, S. M., Mostafizur, A. B. M. & Saha, A. Diversity of cropping pattern in Bogra. *Bangl. Rice J.* **21**, 73-90 (2017).
36. FAO. *FAO Rice Information* (FAO, Rome, Italy, 2002).
37. Brazilian Institute of Geography and Statistics. Census of Agriculture. (accessed 2 August 2021); http://censos.ibge.gov.br.
38. Heinemann, A. B., Ramirez-Villegas, J., Rebolledo, M. C., Neto, G. M. F. C. & Castro, A. P. Upland rice breeding led to increased drought sensitivity in Brazil. *Field Crops Res.* **231**, 57-67 (2019).
39. National Bureau of Statistics. National data. Crop harvested area. (accessed 2 August 2021); http://www.stats.gov.cn.
40. USDA. Foreign agriculture service. Crop production maps. (accessed 2 August 2021); http://ipad.fas.usda.gov/rssiws/al/na_cropprod.aspx.
41. Hasan, E. Proposing mitigation strategies for reducing the impact of rice cultivation on climate change in Egypt. *Water Sci.* **27**, 69-77 (2013).
42. Center for Data and Information Systems Ministry of Agriculture of Indonesia. Agricultural Statistics Database. (accessed 2 August 2021); http://aplikasi2.pertanian.go.id/bdsp/id/home.

43. International Rice Research Institute. *IRRI strategic assessment (Unpublished dataset)* (International Rice Research Institute, Los Baños, Philippines, 2013).
44. Government of India. *Agricultural Statistics at a glance* (Government of India Controller of Publications, Delhi, India, 2018).
45. Dwivedi, B. S., Singh, V. K. & Meena, M. C. *Efficient Nitrogen Management Under Predominant Cropping Systems of India. The Indian nitrogen assessment: Sources of reactive nitrogen, environmental and climate effects, management options, and policies* (Woodhead Publishing, Cambridge, USA and Kidlington, United Kingdom, 2017).
46. National Statistics Institute of Mali. Mali Data Portal. Socio-Economic Database. (accessed 2 August 2021); http://www.instat-mali.org.
47. Central Statistical Organization. Myanmar Statistical Yearbook. (accessed 2 August 2021); http://www.csostat.gov.mm.
48. Mataia, A. B., Beltran, J. C., Manalili, R. G., Catudan, B. M., Francisco, N. M. & Flores, A. C. Rice value chain analysis in the Philippines: Value Addition, Constraints, and Upgrading Strategies. *Asian J. Agr. Dev.* **17**, 19-42 (2020).
49. Office of Agricultural Economics. Agricultural statistics. Agricultural Information Center. (accessed 2 August 2021); http://www.oae.go.th.
50. Department of Agricultural and Resource Economics. Rice Economy of Thailand. Agricultural and Resource Economics Working Paper No. 2562/1 (Kasetsart University, Bangkok, Thailand, 2019).
51. US Department of Agriculture-National Agricultural Statistics Service. Quickstats2.0. (accessed 1 August 2021); http://quickstats.nass.usda.gov/.
52. Tseng, M. C. et al. Field-level factors for closing yield gaps in high-yielding rice systems of Uruguay. *Field Crops Res.* **264**, 108097 (2021).
53. Vietnam General Statstics Office of Vietnam. Statistical data. (accessed 2 August 2021); http://www.gso.gov.vn/en/statistical-data.
54. Arai, H., Hosen, Y., Chiem, N. H. & Inubushi, K. Alternate wetting and drying enhanced the yield of a triple-cropping rice paddy of the Mekong Delta. *Soil Sci. Plant Nutr.* 1-14 (2021).
55. Espe, M. B. et al. Yield gap analysis of US rice production systems shows opportunities for improvement. *Field Crops Res.* **196**, 276-283 (2016).

56. Espe, M. B., Yang, H., Cassman, K. G., Guilpart, N., Sharifi, H. & Linquist, B. A. Estimating yield potential in temperate high-yielding, direct-seeded US rice production systems. *Field Crops Res.* **193**, 123-132 (2016).
57. Espino, L. A., Mutters, R. R., Buttner P., Klonsky, K., Stewart, D. & Tumber, K. P. *Sample costs to produce rice* (University of California Cooperative Extension, Division of Agriculture and Natural resources and Department of Agriculture and Resource Economics, Davis, CA, USA, 2016).
58. Livezey, J. & Foreman, L. *Characteristics and Production Costs of U.S. Rice Farms. Statistical Bulletin No. 974-7* (USDA, Washington, DC, USA, 2004).
59. Adams, K. J. Irrigation Practice Adoption: Causes and Consequences in the Arkansas Delta. Theses and Dissertations. 2763 (2018).
60. Texas County Extension. *Rice Cost of Production Estimates for the 2007 Crop* (Texas County Extension, Texas, USA, 2007)
61. University of California Cooperative Extension, Division of Agriculture and Natural resources and Department of Agriculture and Resource Economics. Water Use by Rice. (accessed 1 August 2021); http://rice.ucanr.edu/Water_Use_by_Rice.
62. Tseng, M. C. et al. Towards actionable research frameworks for sustainable intensification in high-yielding rice systems. *Sci. Rep.* **10**, 1-13 (2020).
63. Hareau, G. G., Mills, B. F. & Norton, G. W. The potential benefits of herbicide-resistant transgenic rice in Uruguay: Lessons for small developing countries. *Food Policy* **31**, 162-179 (2006).
64. Alaimo, V., Bosch, M., Gualavisí, M. & Villa, J. M. *Measuring the Cost of Salaried Labor in Latin America and the Caribbean. IDB Technical Note 1291* (Inter-American Development Bank, Washington, DC, USA, 2017).
65. Riccetto, S., Capurro, M. C. & Alvaro, R. Strategies to minimize water consumption while maintaining productivity in Uruguayan rice production. *Agrociencia (Montevideo)* **21**, 109-119 (2017).
66. Carracelas, G., Hornbuckle, J., Rosas, J. & Roel, A. Irrigation management strategies to increase water productivity in Oryza sativa (rice) in Uruguay. *Agr. Water Manage.* **222**, 161-172 (2019).

67. Ribas, G. G. et al. Assessing yield and economic impact of introducing soybean to the lowland rice system in southern Brazil. *Agric. Syst.* **188**, 103036 (2021).
68. Ribas, G. G. et al. Assessing factors related to yield gaps in flooded rice in Southern Brazil. *Agron. J.* **113**, 3341-3350 (2021).
69. García, R. R. M. et al. Assessing the sustainability of rice production in Brazil and Cuba. J. Agr. *Food Res.* **4**, 100152 (2021).
70. Coltro, L., Marton, L. F. M., Pilecco, F. P., Pilecco, A. C. & Mattei, L. F. Environmental profile of rice production in Southern Brazil: A comparison between irrigated and subsurface drip irrigated cropping systems. *J. Clean. Prod.* **153**, 491-505 (2017).
71. Debnath, S., Mishra, A., Mailapalli, D. R., Raghuwanshi, N. S. & Sridhar, V. Assessment of rice yield gap under a changing climate in India. *J. Water Clim. Change* **12**, 1245-1267 (2021).
72. Moya, P. F. et al. *The economics of intensively irrigated rice in Asia. Increasing productivity of intensive rice systems through site-specific nutrient management* (Science Publishers Inc. and International Rice Research Institute, Enfield NH, USA and Los Baños, Philippines, 2004).
73. Abrol, Y. P. et al. *The Indian nitrogen assessment: Sources of reactive nitrogen, environmental and climate effects, management options, and policies* (Woodhead Publishing, Cambridge, USA and Kidlington, United Kingdom, 2017).
74. Reddy, D. N. & Venkatanarayan, M. *Declining labour use in agriculture: A case of rice cultivation in Andhra Pradesh. MPRA Paper 4920421* (National Institute of Rural Development, Hyderabad, India, 2013)
75. Kar, I., Mishra, A., Behera, B., Khanda, C., Kumar, V. & Kumar, A. Productivity trade-off with different water regimes and genotypes of rice under non-puddled conditions in Eastern India. *Field Crops Res.* **222**, 218-229 (2018).
76. Mahajan, G., Bharaj, T. S. & Timsina, J. Yield and water productivity of rice as affected by time of transplanting in Punjab, India. *Agr. Water Manage.* **96**, 525-532 (2009).
77. National Bureau of Statistics of China. National Data, Crop yield. (accessed 1 August 2021); http://data.stats.gov.cn/easyquery.htm?cn=C01.
78. Cui, Z. et al. Pursuing sustainable productivity with millions of smallholder farmers. *Nature* **555**, 363-366 (2018).

79. National Development and Reform Commission of China. *National Agricultural Products Cost-Benefit Data Compilation* (China Statistics Press, Beijing, China, 2018).
80. Chen, X. et al. Producing more grain with lower environmental costs. *Nature*, **514**, 486-489 (2014).
81. Zhang, C., Shi G. M., Shen, J. & Hu, R. F. Productivity effect and overuse of pesticide in crop production in China. *J. Integr. Agr.* **14**, 1903-1910. (2015).
82. Yuan, S., Cassman, K. G., Huang, J., Peng, S. & Grassini, P. Can ratoon cropping improve resource use efficiencies and profitability of rice in central China?. *Field Crops Res.* **234**, 66-72 (2019).
83. Cabangon, R. J. et al. Effect of irrigation method and N-fertilizer management on rice yield, water productivity and nutrient-use efficiencies in typical lowland rice conditions in China. *Paddy Water Environ.* **2**, 195-206 (2004).
84. Xu, J., Peng, S., Yang, S. & Wang, W. Ammonia volatilization losses from a rice paddy with different irrigation and nitrogen managements. *Agr. Water Manage.* **104**, 184-192 (2012).
85. Shao, G. C., Deng, S., Liu, N., Yu, S. E., Wang, M. H. & She, D. L. Effects of controlled irrigation and drainage on growth, grain yield and water use in paddy rice. *Eur. J. Agron.* **53**, 1-9 (2014).
86. Devkota, K. P. et al. Economic and environmental indicators of sustainable rice cultivation: a comparison across intensive irrigated rice cropping systems in six Asian countries. *Ecol. Indic.* **105**, 199-214 (2019).
87. Stuart, A. M. et al. Yield gaps in rice-based farming systems: Insights from local studies and prospects for future analysis. *Field Crops Res.* **194**, 43-56 (2016).
88. Matsuda, M. Intensification level of rice farming in Myanmar: implication for its sustainable development. *Environ. Dev. Sustain.* **13**, 51-64 (2011).
89. Laborte, A. G., de Bie, K. C., Smaling, E. M., Moya, P. F., Boling, A. A. & Van Ittersum, M. K. Rice yields and yield gaps in Southeast Asia: past trends and future outlook. *Eur. J. Agron.* **36**, 9-20 (2012).
90. Linn, T. & Maenhout, B. Measuring the efficiency of rice production in Myanmar using data envelopment analysis. *Asian J. Agr. Dev.* **16**, 1-24 (2019).

91. Stuart, A. M. et al. The application of best management practices increases the profitability and sustainability of rice farming in the central plains of Thailand. *Field Crops Res.* **220**, 78-87 (2018).
92. Satawathananont, S., Chatuporn, S., Niyomvit, L., Kongchum, M., Sookthongsa, J. & Dobermann, A. *Site-specific nutrient management in irrigated rice systems of Central Thailand. Increasing productivity of intensive rice systems through site-specific nutrient management* (Science Publishers Inc. and International Rice Research Institute, Enfield NH, USA and Los Baños, Philippines, 2004).
93. Ullah, H., Giri, S., Attia, A. & Datta, A. Effects of establishment method and water management on yield and water productivity of tropical lowland rice. *Exp. Agr.* **56**, 331-346 (2020).
94. Maneepitak, S., Ullah, H., Paothong, K., Kachenchart, B., Datta, A. & Shrestha, R. P. Effect of water and rice straw management practices on yield and water productivity of irrigated lowland rice in the Central Plain of Thailand. *Agr. Water Manage.* **211**, 89-97 (2019).
95. Sriphirom, P., Chidthaisong, A. & Towprayoon, S. Effect of alternate wetting and drying water management on rice cultivation with low emissions and low water used during wet and dry season. *J. Clean. Prod.* **223**, 980-988 (2019).
96. Tan, P. S. et al. *Site-specific nutrient management in irrigated rice systems of the Mekong Delta of Vietnam. Increasing productivity of intensive rice systems through site-specific nutrient management* (Science Publishers Inc. and International Rice Research Institute, Enfield NH, USA and Los Baños, Philippines, 2004).
97. Stuart, A. M. et al. On-farm assessment of different rice crop management practices in the Mekong Delta, Vietnam, using sustainability performance indicators. *Field Crops Res.* **229**, 103-114 (2018).
98. Abdulrahman, S., Susanti, Z., Pahim, A. D., Dobermann, A. & Witt, C. *Site-specific nutrient management in intensive irrigated rice systems of West Java, Indonesia. Increasing productivity of intensive rice systems through site-specific nutrient management* (Science Publishers Inc. and International Rice Research Institute, Enfield NH, USA and Los Baños, Philippines, 2004).
99. Agus, F. et al. Yield gaps in intensive rice-maize cropping sequences in the humid tropics of Indonesia. *Field Crops Res.* **237**, 12-22 (2019).

100. Silva, J. V., Reidsma, P., Laborte, A. G. & Van Ittersum, M. K. Explaining rice yields and yield gaps in Central Luzon, Philippines: An application of stochastic frontier analysis and crop modelling. *Eur. J. Agron.* **82**, 223-241 (2017).
101. Moya, P., Kajisa, K., Barker, R., Mohanty, S., Gascon, F. & San Valentin, M. R. *Changes in rice farming in the Philippines: Insights from five decades of a household-level survey* (International Rice Research Institute, Los Baños, Philippines, 2015).
102. Gines, H. C., Redondo, G. O., Estigoy, A. P. & Dobermann, A. *Site-specific nutrient management in irrigated rice systems of Central Luzon, Philippines. Increasing productivity of intensive rice systems through site-specific nutrient management* (Science Publishers Inc. and International Rice Research Institute, Enfield NH, USA and Los Baños, Philippines, 2004).
103. Magahud, J. C., Dalumpines, S. L. P., Sanchez, P. B. & Collado, W. B. Extent and determining factors of fertilizer applications and rice straw management in major irrigated rice areas of the Philippines. *Philipp. Sci. Lett.* **13**, 61-69 (2020).
104. Tabbal, D. F., Bouman, B. A. M., Bhuiyan, S. I., Sibayan, E. B. & Sattar, M. A. On-farm strategies for reducing water input in irrigated rice; case studies in the Philippines. *Agr. Water Manage.* **56**, 93-112 (2002).
105. Belder, P., Spiertz, J. H. J., Bouman, B. A. M., Lu, G. & Tuong, T. P. Nitrogen economy and water productivity of lowland rice under water-saving irrigation. *Field Crops Res.* **93**, 169-185 (2005).
106. Lampayan, R. M. et al. Effects of seedbed management and delayed transplanting of rice seedlings on crop performance, grain yield, and water productivity. *Field Crops Res.* **183**, 303-314 (2015).
107. Dyer, J. A. & Desjardins, R. L. Carbon dioxide emissions associated with the manufacturing of tractors and farm machinery in Canada. *Biosyst. Eng.* **93,** 107-118 (2006).
108. Khoshnevisan, B., Rafiee, S., Omid, M., Yousefi, M. & Movahedi, M. Modeling of energy consumption and GHG (greenhouse gas) emissions in wheat production in Esfahan province of Iran using artificial neural networks. *Energy* **52,** 333-338 (2013).
109. Pishgar-Komleh, S. H., Omid, M. & Heidari, M. D. On the study of energy use and GHG (greenhouse gas) emissions in greenhouse cucumber production in Yazd province. *Energy* **59,** 63-71 (2013).

110. Casey, J. W. & Holden, N. M. Analysis of greenhouse gas emissions from the average Irish milk production system. *Agric. Syst.* **86,** 97-114 (2005).
111. Mondani, F., Aleagha, S., Khoramivafa, M. & Ghobadi, R. Evaluation of greenhouse gases emission based on energy consumption in wheat agroecosystems. *Energy Rep.* **3,** 37-45 (2017).
112. Lal, R. Carbon emission from farm operations. *Environ. Int.* **30,** 981-990 (2004).
113. Pathak, H. & Wassmann, R. Introducing greenhouse gas mitigation as a development objective in rice-based agriculture: I. Generation of technical coefficients. *Agric. Syst.* **94,** 807-825 (2007).
114. Li, X. M. & Kotelko, M. *An integrated manure utilization system (IMUS): its social and environmental benefits. The 3rd international methane and nitrous oxide mitigation conference, Lecture No.: AG056* (Beijing, China, 2003).
115. Yang, X., Gao, W., Zhang, M., Chen, Y. & Sui, P. Reducing agricultural carbon footprint through diversified crop rotation systems in the North China Plain. *J. Clean. Prod.* **76,** 131-139 (2014).
116. Guo, J., Song, Z., Zhu, Y., Wei, W., Li, S. & Yu, Y. The characteristics of yield-scaled methane emission from paddy field in recent 35-year in China: A meta-analysis. *J. Clean. Prod.* **161,** 1044-1050 (2017).
117. Hokazono, S. & Hayashi, K. Variability in environmental impacts during conversion from conventional to organic farming: a comparison among three rice production systems in Japan. *J. Clean. Prod.* **28,** 101-112 (2012).
118. Fusi, A., Bacenetti, J., González-García, S., Vercesi, A., Bocchi, S. & Fiala, M. Environmental profile of paddy rice cultivation with different straw management. *Sci. Total Environ.* **494,** 119-128 (2014).
119. Harada, H., Kobayashi, H. & Shindo, H. Reduction in greenhouse gas emissions by no-tilling rice cultivation in Hachirogata polder, northern Japan: Life-cycle inventory analysis. *Soil Sci. Plant Nutr.* **53,** 668-677 (2007).
120. Mikkola, H. J. & Ahokas, J. Indirect energy input of agricultural machinery in bioenergy production. *Renew. Energy* **35,** 23-28 (2010).
121. Alluvione, F., Moretti, B., Sacco, D. & Grignani, C. EUE (energy use efficiency) of cropping systems for a sustainable agriculture. *Energy* **36,** 4468-4481 (2011).

122. Arizpe, N., Giampietro, M. & Ramos-Martin, J. Food security and fossil energy dependence: An international comparison of the use of fossil energy in agriculture (1991-2003). *Crit. Rev. Plant Sci.* **30,** 45-63 (2011).
123. IEA. *Chemical and petrochemical industry. Tracking Industrial Energy Efficiency and CO_2 Emissions* (IEA, Paris, France, 2007).
124. Meul, M., Nevens, F., Reheul, D. & Hofman, G. Energy use efficiency of specialized dairy, arable and pig farms in Flanders. *Agric. Ecosyst. Environ.* **119,** 135-144 (2007).
125. Wu, H. et al. Temporal trends and spatial patterns of energy use efficiency and greenhouse gas emissions in crop production of Anhui Province, China. *Energy* **133,** 955-968 (2017).
126. Lal, B. et al. Input-output energy analysis of rainfed rice-based cropping systems in Eastern India. *Agron. J.* **107,** 1750-1756 (2015).
127. West, T. O. & Marland, G. A synthesis of carbon sequestration, carbon emissions, and net carbon flux in agriculture: comparing tillage practices in the United States. *Agr. Ecosyst. Environ.* **91,** 217-232 (2002).
128. EIA. Annual Energy Review. Energy consumption, expenditures, and emissions indicators estimates. (accessed 10 October 2020); www.eia.gov/totalenergy/data/annual/.
129. Zhang, W. et al. New technologies reduce greenhouse gas emissions from nitrogenous fertilizer in China. *Proc. Natl Acad. Sci. USA* **110,** 8375-8380 (2013).
130. Singh, P., Singh, G. & Sodhi, G. P. Energy auditing and optimization approach for improving energy efficiency of rice cultivation in south-western Punjab, India. *Energy* **174,** 269-279 (2019).
131. Xie, F. & Hardy, B. *Accelerating hybrid rice development* (International Rice Research Institute, Los Baños, Philippines, 2009).
132. Peng, S. Dilemma and way-out of hybrid rice during the transition period in China. *Acta. Agron. Sin.* **42,** 313-319 (2016).
