# Supplementary material for: Sustainable intensification for a larger global rice bowl
Source: Nat Commun. 2021 Dec 9;12:7163. doi: 10.1038/s41467-021-27424-z (PMC8660894; doi:10.1038/s41467-021-27424-z)
Supplement: Supplementary file 2 — Reporting Summary [file 41467_2021_27424_MOESM2_ESM.pdf]

nature research

Corresponding author(s): Patricio Grassini

Last updated by author(s): Nov 11, 2021

Reporting Summary

Nature Research wishes to improve the reproducibility of the work that we publish. This form provides structure for consistency and transparency in reporting. For further information on Nature Research policies, see our Editorial Policies and the Editorial Policy Checklist.

Statistics

For all statistical analyses, confirm that the following items are present in the figure legend, table legend, main text, or Methods section.

n/a	Confirmed	
☐	☒	The exact sample size (*n*) for each experimental group/condition, given as a discrete number and unit of measurement
☐	☒	A statement on whether measurements were taken from distinct samples or whether the same sample was measured repeatedly
☐	☒	The statistical test(s) used AND whether they are one- or two-sided<br>*Only common tests should be described solely by name; describe more complex techniques in the Methods section.*
☒	☐	A description of all covariates tested
☒	☐	A description of any assumptions or corrections, such as tests of normality and adjustment for multiple comparisons
☒	☐	A full description of the statistical parameters including central tendency (e.g. means) or other basic estimates (e.g. regression coefficient) AND variation (e.g. standard deviation) or associated estimates of uncertainty (e.g. confidence intervals)
☐	☒	For null hypothesis testing, the test statistic (e.g. *F*, *t*, *r*) with confidence intervals, effect sizes, degrees of freedom and *P* value noted<br>*Give P values as exact values whenever suitable.*
☒	☐	For Bayesian analysis, information on the choice of priors and Markov chain Monte Carlo settings
☒	☐	For hierarchical and complex designs, identification of the appropriate level for tests and full reporting of outcomes
☐	☒	Estimates of effect sizes (e.g. Cohen's *d*, Pearson's *r*), indicating how they were calculated

Our web collection on statistics for biologists contains articles on many of the points above.

Software and code

Policy information about availability of computer code

Data collection	Data on actual yields, crop calendars, applied inputs, and dominate crop management practices were provided by agricultural specialists.
Data analysis	Crop growth and development model ORYZA2000 or ORYZA (v3) and APSIM in the case of India were used to simulate yield potential. Statistix 8, SigmaPlot 12.5 were used for statistical analysis. ArcGIS 10.5 was used to create maps.

For manuscripts utilizing custom algorithms or software that are central to the research but not yet described in published literature, software must be made available to editors and reviewers. We strongly encourage code deposition in a community repository (e.g. GitHub). See the Nature Research guidelines for submitting code & software for further information.

Data

Policy information about availability of data

All manuscripts must include a data availability statement. This statement should provide the following information, where applicable:

- Accession codes, unique identifiers, or web links for publicly available datasets
- A list of figures that have associated raw data
- A description of any restrictions on data availability

Data on yield potential from Global Yield Gap Atlas are available at www.yieldgap.org. Data on per-capita gross domestic product from the World Bank are available at https://databank.worldbank.org. Data on rice distribution from SPAM are available at www.mapspam.info. Data on rice area and production from FAOSTAT are available at www.fao.org/faostat. We note that questionnaire participants gave full consent to share the data used in our study. The source data are provided with this paper.

Field-specific reporting

Please select the one below that is the best fit for your research. If you are not sure, read the appropriate sections before making your selection.

☐ Life sciences ☐ Behavioural & social sciences ☒ Ecological, evolutionary & environmental sciences

For a reference copy of the document with all sections, see nature.com/documents/nr-reporting-summary-flat.pdf

Ecological, evolutionary & environmental sciences study design

All studies must disclose on these points even when the disclosure is negative.

Study description	We assessed yield gap, resource-use efficiency (including water, pesticides, nitrogen, labor, and energy), and environmental impact across 32 rice cropping systems, together accounting for 88% of global rice production.
Research sample	We followed two steps to select the dominant cropping systems in each country. Within each country, our study focused on the main rice producing area(s). Across the 18 countries, this study included a total of 32 rice cropping systems, which, in turn, covered 51% of the global rice harvested area. Data on actual yields, crop calendars, applied inputs, and dominate crop management practices were provided by agricultural specialists. Data on yield potential from Global Yield Gap Atlas are available at www.yieldgap.org. Data on per-capita gross domestic product from the World Bank are available at https://databank.worldbank.org. Data on rice distribution from SPAM are available at www.mapspam.info. Data on rice area and production from FAOSTAT are available at www.fao.org/faostat. Data on energy and emission factors are retrieved from publishes studies.
Sampling strategy	Eighteen rice-producing countries were selected for our analysis. Those countries account for 88% and 86% of global rice production and harvested rice area, respectively. We followed two steps to select the dominant cropping systems in each country. Within each country, our study focused on the main rice producing area(s). Across the 18 countries, this study included a total of 32 rice cropping systems, which, in turn, covered 51% of the global rice harvested area. The 32 systems encompassed a wide range of biophysical and socio-economic context. Agronomic information for each rice cycle in each of the 32 cropping systems was collected via structure questionnaires completed by agricultural specialists in each country or region. Rice grain yield was reported for, at least, three recent cropping seasons for each rice cycle in each cropping system.
Data collection	Data on yield potential, per-capita gross domestic product, rice distribution, rice area and production, and energy and emission factors were downloaded from Global Yield Gap Atlas, the World Bank, SPAM, FAOSTAT, and previous publications. Data on actual yield, applied inputs, and dominate crop management practices were collected via structure questionnaires in Microsoft Excel 2016 completed by agricultural specialists in each country or region. Average values for each cropping system reported by country experts were retrieved from survey data available from previous projects.
Timing and spatial scale	The evaluation of yield gap, resource-use efficiency, and environmental impact was performed across 32 rice cropping systems in 18 countries at both per rice cycle and per year basis. Yield potential corresponded to the average value estimated in the Global Yield Gap Atlas using weather data for at least most recent 10 years. Data on national average per-capita gross domestic product, total rice area, and total rice production were the average from 2015-2017. Data on applied inputs and dominate crop management practices for each rice cycle in each cropping system were the average from 2015-2017. Data on actual yield for each rice cycle in each cropping system were collected for, at least, three recent cropping seasons.
Data exclusions	No data were excluded from the analysis.
Reproducibility	Data used in this study are available within the paper [and its supplementary information files]. All attempts at replication were successful.
Randomization	We purposely selected cropping systems that account for largest share of global rice production and portray a wide range of biophysical and socio-economic backgrounds. Management data were collected via experts in each country from those areas that were representative of the main rice production systems in each country.
Blinding	Not applicable as we do not see any step in our study In which co-authors or experts collecting data and/or performing the analysis were prevented from knowing certain information that may somehow influence their data collection and/or analysis.

Did the study involve field work? ☐ Yes ☒ No

Reporting for specific materials, systems and methods

We require information from authors about some types of materials, experimental systems and methods used in many studies. Here, indicate whether each material, system or method listed is relevant to your study. If you are not sure if a list item applies to your research, read the appropriate section before selecting a response.

Materials & experimental systems

n/a	Involved in the study
☒	☐ Antibodies
☒	☐ Eukaryotic cell lines
☒	☐ Palaeontology and archaeology
☒	☐ Animals and other organisms
☒	☐ Human research participants
☒	☐ Clinical data
☒	☐ Dual use research of concern

Methods

n/a	Involved in the study
☒	☐ ChIP-seq
☒	☐ Flow cytometry
☒	☐ MRI-based neuroimaging
